# Alternative pre-mRNA Splicing and Gene Expression Patterns in Midbrain Lineage Cells Carrying Familial Parkinson’s Disease Mutations

**DOI:** 10.1101/2024.02.28.582420

**Published:** 2025-09-04

**Authors:** Yeon J. Lee, Khaja Syed, Oriol Busquets, Hanqin Li, Jesse Dunnack, Helen S. Bateup, Frank Soldner, Dirk Hockemeyer, Donald C. Rio

**Affiliations:** 1 University of California, Berkeley, Molecular and Cell Biology, Berkeley, CA, 94720; 2 University of California, Berkeley, California Institute for Quantitative Biosciences, Berkeley, CA, 94720; 3 Dominick P. Purpura Department of Neuroscience, Albert Einstein College of Medicine, Rose F. Kennedy Center, Albert Einstein College of Medicine, 1410 Pelham Parkway South, Bronx, NY 10461; 4 Department of Genetics, Albert Einstein College of Medicine, 1301 Morris Park Ave., Bronx, NY 10461; 5 Ruth L. and David S. Gottesman Institute for Stem Cell and Regenerative Medicine Research, Albert Einstein College of Medicine, 1301 Morris Park Ave., Bronx, NY 10461; 6 University of California, Berkeley, Department of Neuroscience, Berkeley, CA 94720; 7 Helen Wills Neuroscience Institute, University of California, Berkeley, Berkeley, CA 94720; 8 Innovative Genomics Institute, University of California, Berkeley, CA, 94720; 9 Ruth L. and David S. Gottesman Institute for Stem Cell and Regenerative Medicine Research, Albert Einstein College of Medicine, 1301 Morris Park Ave., Bronx, NY 10461; 10 Aligning Science Across Parkinson’s (ASAP) Collaborative Research Network, Chevy Chase, MD, 20815.

## Abstract

Parkinson's disease (PD) arises from genetic and environmental factors. Human genetics has identified mutations in ~20 inherited familial genes linked to monogenic forms of PD. To investigate the effects of individual familial PD mutations, human pluripotent embryonic stem cells (hPSCs) carrying 12 distinct familial PD mutations were differentiated into midbrain lineage cells, including dopaminergic (mDA) neurons. Global gene expression and pre-mRNA splicing patterns were analyzed in midbrain cultures carrying pathogenic PD mutations in the *PRKN*, *SNCA*, *LRRK2*, *PINK1*, *DNAJC6*, *FBXO7*, *SYNJ1*, DJ1, *VPS13C*, *ATP13A2* and *GBA1* genes. This analysis revealed that these familial PD mutations lead to pre-mRNA splicing changes linked to RNA splicing factors and to pathways controlling cell projections, cytoskeleton, GTPase regulation and others. Importantly, we have also shown that subsets of these splicing changes overlap with changes found in PD patient postmortem brains. Mutation-specific pre-mRNA isoforms may function as both diagnostic biomarkers for familial PD-associated genotypes and promising therapeutic targets.

## Introduction

Parkinson’s disease (PD) affects 2–3% of individuals over 65 years of age and is the second most common neurodegenerative disease (10.1101/2024.02.12.579917Aarsland et al., 2021, Poewe et al., 2017). PD is characterized by severe deterioration of motor function caused by loss of dopaminergic neurons in the substantia nigra and is accompanied by deficient dopamine release in the caudate/putamen (Kouli et al., 2018). A hallmark of PD is the intracellular aggregation of the protein α-synuclein into histologically detectable inclusions called Lewy bodies (Aarsland et al., 2021, Poewe et al., 2017). Cells outside the brain, including cells throughout the central and peripheral autonomic nervous system are also affected as the disease progresses (Poewe et al., 2017, Mendoza-Velasquez et al., 2019, Chen et al., 2020).

Lewy body diseases include dementia with Lewy body disease (DLB) and Parkinson's disease with dementia (PDD). In PD, PDD and DLB, the protein α-synuclein abnormally accumulates in the brain in aggregates, or clumps, called Lewy bodies. Key insights into the mechanism of the disease came from human genetic studies that have identified 20 genes to cause familial monogenetically inherited forms of PD (Aarsland et al., 2021, Poewe et al., 2017). Many of these genes, including *SNCA*, which encodes α-synuclein itself, point to defects in the lysosomal-autophagy system (LAS) (Winslow et al., 2010, Navarro-Romero et al., 2020). α-synuclein oligomers inhibit macro-autophagy, contributing to protein accumulation in PD (Winslow and Rubinsztein, 2011, Fellner et al., 2021). Similarly, LRRK2 mutations linked to familial PD impair the function of lysosome-associated membrane protein (LAMP)-positive compartments and cilia (Khan et al., 2021, O'Hara et al., 2020, Alessi and Pfeffer, 2024), potentially by disrupting autophagy within these structures or affecting autophagosome transport (Boecker et al., 2021*)*. Other mutant genes linked to PD affect lysosome or mitochondrial function, as well as lipid and protein sorting (Navarro-Romero et al., 2020, Plotegher and Duchen, 2017). The proposed underlying mechanisms include defects in proteostasis, calcium homeostasis, mitochondrial and autophagy dysfunction, oxidative stress, altered axonal transport and neuroinflammation (Lehtonen et al., 2019, Wilson et al., 2023).

Dysregulated alternative pre-mRNA splicing is emerging as a new and common mechanism implicated in the pathogenesis of neurodegenerative diseases such as Alzheimer's disease, PD and spinal muscular atrophy (SMA) (Nikom and Zheng, 2023, Li et al., 2021, Rogalska et al., 2023). We have developed a platform to study the effects of familial, hereditary PD mutations on pre-mRNA splicing patterns and gene expression changes in *in vitro* cultured human embryonic stem cell (hPSC)-derived dopaminergic (DA) neurons. We have previously developed a genome-editing platform using Cas9 and prime editing to engineer many of the most common familial PD mutations into hPSCs (Li et al., 2022b, Busquets et al., 2025). Using cells from this resource, we examined the effects on gene expression and pre-mRNA splicing patterns caused by common familial PD mutations in the *PRKN, SNCA, LRRK2, PINK1, DNAJC6, FBXO7, SYNJ1, PARK7, VPS13C, ATP13A2 and GBA1* genes (Kruger et al., 1998, Polymeropoulos et al., 1997, Isaacs et al., 2017, Sasaki et al., 2004). Following differentiation of multiple independent cell clones carrying these mutations, we carried out deep bulk short-read RNA sequencing (RNA-seq) using the Illumina platform to determine pre-mRNA patterns, using an annotation-free method called the Junction Usage Model (JUM) for comparison of splicing patterns (Wang and Rio, 2018) between the mutant and edited wild type (EWT) control cells (cells that received editing reagents in parallel but did not create the desired mutations). We found that cells carrying the 12 familial PD mutations exhibited many changes in spliced transcript isoforms from pathways governing cell projections, the cytoskeleton and GTPase regulation and sometimes impacting splicing factors themselves. There were also changes at the level of gene expression with different degrees of overlap with genes whose splicing patterns changed depending on the mutation. Moreover, comparison of our RNA-seq data to PD patient postmortem brain tissue RNA-seq data (Feleke et al., 2021) from patients with PD and related synucleinopathies including PD with dementia (PDD) and dementia with Lewy Bodies (DLB). Taken together, our data supports the idea that the pre-mRNA splicing pattern defects we observe in hPSC-derived midbrain DA neurons (mDA) *in vitro* are relevant to PD, that these distinct transcript isoforms should serve as useful biomarkers and might be amenable to RNA therapeutics (Anthony, 2022, Li et al., 2022a, Rogalska et al., 2023).

## Results

### Differentiation of dopaminergic (DA) neurons from human embryonic stem cells (hPSCs) carrying familial PD mutations.

**Figure 1.**
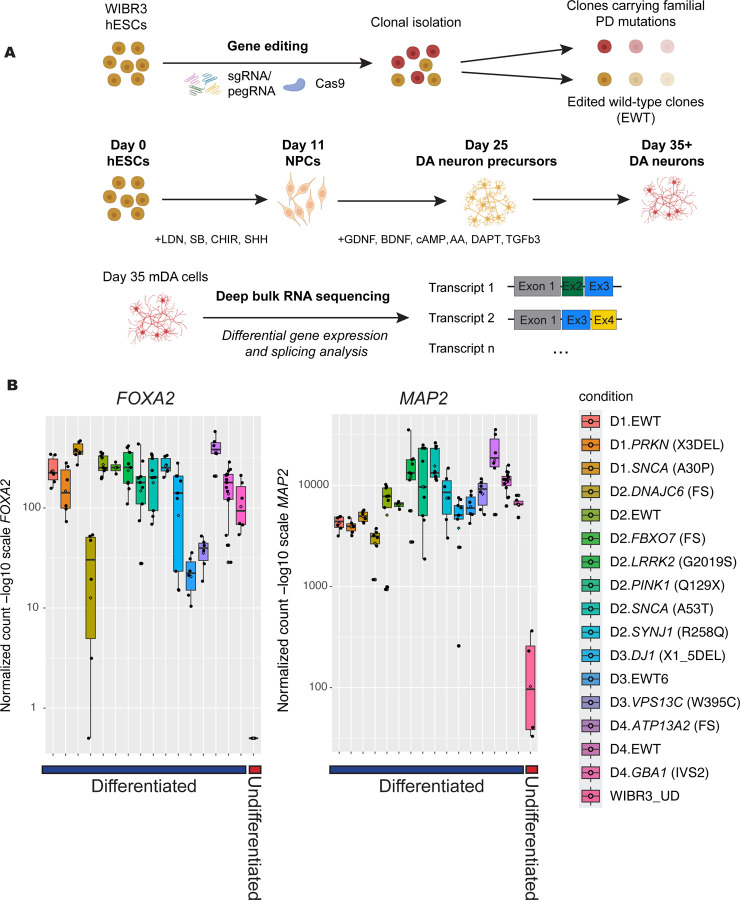
Platform for the medium-throughput genome-editing of human embryonic stem cells (hPSCs) carrying relevant familial Parkinson’s mutations and differentiation of these cells to midbrain lineage cells. A. Schematic diagram of editing pipeline and expansion into clonal hPSC lines. B. Normalized gene counts for neuronal markers including *FOXA2* (*forkhead box A2*) and *MAP2* (*microtubule-associated protein 2*), in 35d differentiated edited wild-type (EWT), familial *PRKN, SNCA, LRRK2, PINK1, DNAJC6, FBX7, SYNJ1,* DJ1*, VPS13C, ATP13A2 and GBA1* mutant cells, in comparison to undifferentiated WIBR3 hPSCs.

To study the effects of PD-linked mutations on splicing and gene expression, we differentiated a subset of the Isogenic Stem Cell Collection to Research Parkinson’s Disease (iSCORE-PD) collection (Busquets et al., 2025) into mDA. Multiple clonally independent isogenic stem cell lines were generated carrying common familial PD mutations including *PRKN* (X3DEL), *SNCA* (A30P and A53T)*, LRRK2* (G2019S)*, PINK1* (Q129X)*, DNAJC6* (FS)*, FBXO7* (FS)*, SYNJ1* (R258Q)*, PARK7* (X1_5DEL)*, VPS13C* (W395C)*, ATP13A2* (FS) *and GBA1* (IVS2) using CRISPR Cas9 or prime editing techniques, as previously reported (Figure 1A) (Li et al., 2022b, Busquets et al., 2025). hPSCs were differentiated into mDA, which are the primary cell type that degenerates in PD using a modified version of a reported protocol (Kim et al., 2021), which generated dopamine neurons from hPSCs by boosting WNT signaling during early midbrain lineage patterning. After the initial patterning for 11 days, cells were differentiated over 3 weeks. The resulting differentiated mDA cells robustly expressed the key differentiation markers *FOXA2* and *MAP2*, across subclones of familial PD mutant-expressing cells (Figure 1B;(Busquets et al., 2025)). Gene expression analysis from bulk RNA seq data of the differentiated mDA cells at day 35 confirmed the expression of key neuronal markers (e.g. *FOXA2* and *MAP2*) compared to undifferentiated WIBR3 hPSC cells, indicating robust differentiation within these hPSC-derived cell lines expressing familial PD mutations (Figure 1B).

### Identification of unique splicing signatures in hPSC-derived dopaminergic neurons carrying PD-associated mutations

During neuronal differentiation, alternative splicing can modulate diverse processes such as signaling activity, centriolar dynamics and metabolic pathways (Su et al., 2018). RNA-seq data can provide insights into the regulation of alternative splicing by revealing the usage of known or unknown (unannotated) splice sites as well as the expression level of exons. Using 35 day-differentiated mDA cells carrying common familial PD mutations in the *PRKN, SNCA, LRRK2, PINK1, DNAJC6, FBXO7, SYNJ1, PARK7, VPS13C, ATP13A2 and GBA1* genes or wild-type genotypes, we prepared total RNA, poly(A)-mRNA selected and then generated random-primed cDNA libraries from 1–3 independently derived subclonal cell lines in technical triplicates. These libraries were sequenced on the Illumina NovaSeq S1 or S4 platform with between 200–300M paired-end 150bp (PE150) reads. The large datasets coming from this experiment were analyzed using the JUM software (Wang and Rio, 2018) to compare and quantitate pre-mRNA splicing pattern differences between the mutant and edited wild type (EWT) control cells. The ratio between reads including or excluding exons, also known as the percent-spliced-in index (PSI), indicates how efficiently sequences of interest are spliced into mature transcripts. We observed thousands of splicing events that differed between the EWT and mutant cell lines with the PSI cut-off set to > 5%, in canonical splicing patterns and in a more complex category that JUM denotes as “composite”, which is a combination of splicing patterns beyond a simple splicing event within the same splicing structure (Wang and Rio, 2018). RNA-seq data derived from 12 different familial mutant cell lines were analyzed for RNA splicing and gene expression changes compared to EWT controls (Supplemental Tables 1–3). We also compared the splicing signatures for each PD-associated mutation to a published bulk PD patient biopsy tissue RNA-sequencing datasets containing 24 postmortem human brain cortex samples to determine the disease relevance of the observed splicing changes (Feleke et al., 2021).

### PRKN gene exon 3 deletion (X3DEL)

**Figure 2.**
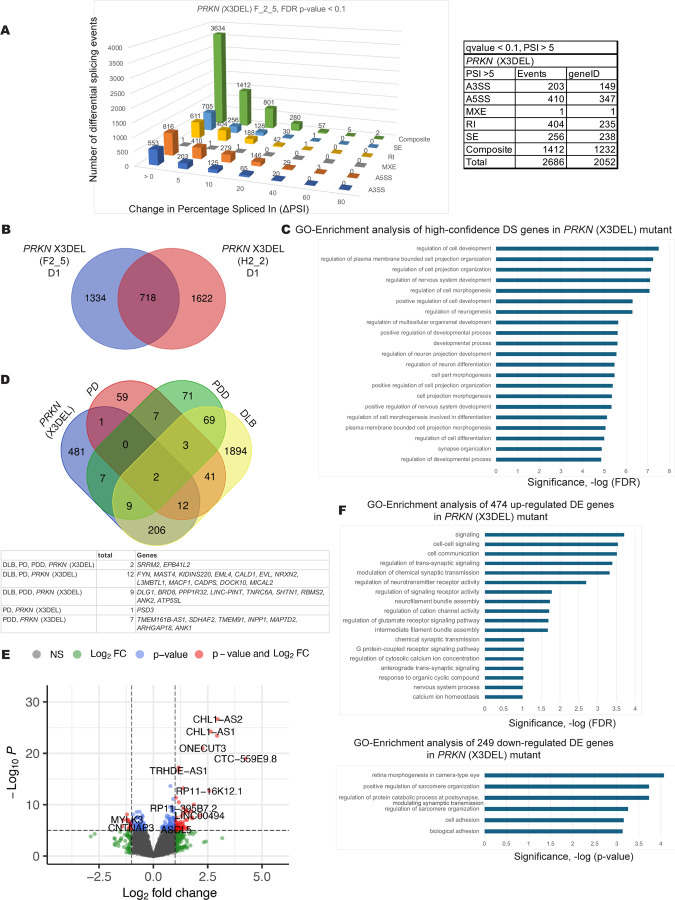
Differentially spliced and differentially expressed transcripts in DA neuronal cells carrying the familial *PRKN* Exon 3 deletion (X3DEL) mutation. A. Differential alternative splicing events detected upon *PRKN* exon 3 deletion in differentiated DA neurons and progenitor cells (35d) using JUM (Wang and Rio, 2018). 718 splicing events were significantly altered in the *PRKN* exon 3 deletion mutant samples in DA neurons and progenitor cells versus the edited wild type control cells (EWT, with adjusted p-value < 0.1, ΔPSI >5). Data for one of three clonal mutant cell lines is shown as an example. Shown on the y-axis is the number of differential splicing events and difference in percentage-spliced-in (or ΔPSI) is shown on the x-axis. The splicing events were filtered based on the magnitude of delta PSI (ΔPSI). B. Comparison of genes that have differentially spliced transcripts in two different clonal lines carrying the *PRKN* (X3DEL) mutation, which results in 718 high confidence differentially spliced gene transcripts. (D1 neuronal differentiation set number). C. A graph of Gene Ontology (GO) term enrichment of 718 differentially spliced transcripts in *PRKN* (X3DEL) mutant DA neuronal cells compared to the edited wild type (EWT) controls. D. Comparison and overlap of differentially spliced transcripts from genes in the *PRKN* (X3DEL) mutant cells to differentially spliced transcripts from genes found in Lewy body disease patient brain biopsy RNA-seq data (Feleke et al., 2021): (Parkinson disease (PD), Parkinson disease with dementia (PDD), dementia with Lewy bodies (DLB)). E. A volcano plot of D1 (differentiation set 1) is shown as an example with some up- and down-regulated gene names shown. DESeq2 expression analysis of the *PRKN* (X3DEL) mutant compared to the edited wild type (EWT) controls reveals 723 differentially expressed genes (adjusted p-values < 0.05, FC > 1.5). F. A graph of Gene Ontology (GO) term enrichment of either 474 up-regulated genes or 249 down-regulated genes in the *PRKN* (X3DEL) mutant protein-expressing mDA neuronal cells compared to the edited wild type (EWT) controls.

PRKN is a ubiquitin ligase acting to ensure mitochondrial quality control. The familial *PRKN* gene mutation (X3DEL) showed many splicing pattern changes (Fig. 2A) and when two mutant clones were compared, 718 high confidence splicing pattern changes were observed compared to edited wild type (EWT) control cell lines (Fig. 2B). This group of transcripts came from genes linked to cell projection organization and neuronal differentiation by Gene Ontology (GO) analyses (Fig. 2C). Comparison of these pre-mRNA splicing changes to RNA-seq data from PD and Lewy Body patient brain biopsies showed overlap for splicing changes in genes linked to PD, PDD and DLB. Notably, DLB had the most splicing changes and overlap with the hPSC-derived *PRKN* mutant splicing changes and the splicing factor *SRRM2* was affected in the mutant hPSCs and all three Lewy body diseases (Fig. 2D). The genes whose transcripts are affected at the level of pre-mRNA splicing are indicated in Supplemental Tables S1 and S2. We also performed differential gene expression profiling using DESeq2 (Love et al., 2014) on *PRKN* X3DEL mutant cells compared to EWT controls and determined a total 723 differentially expressed genes that play roles in the regulation of synaptic signaling and cell adhesion (Fig. 2E and 2F). The differentially expressed genes identified through GO enrichment analysis are detailed in Supplemental Table S3.

### SNCA A30P

**Figure 3.**
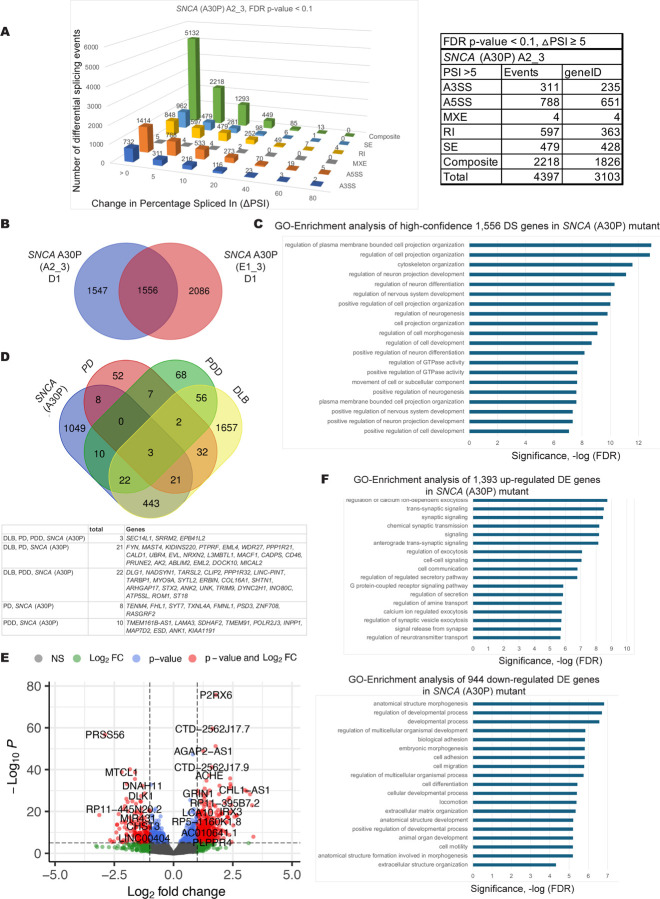
Differentially spliced and differentially expressed transcripts in DA neuronal cells carrying the familial *SNCA* (A30P) mutation. A. Differential alternative splicing events detected upon *SNCA* (A30P) mutation in differentiated DA neurons and progenitor cells (35d) using JUM (Wang and Rio, 2018). 1,566 high confidence splicing events (found in at least two mutant cell clones) were significantly altered in the *SNCA* (A30P) mutant samples in DA neurons and progenitor cells versus the edited wild type controls (EWT; with adjusted p-value < 0.1, ΔPSI ≥5). Data for one of three clonal mutant cell lines is shown as an example. Shown on the y-axis is the number of differential splicing events and differences in percentage-spliced-in (or ΔPSI) is shown on the x-axis. The splicing events were filtered based on the magnitude of delta PSI (ΔPSI). B. Comparison of genes that have differentially spliced transcripts in different clonal lines of the *SNCA* (A30P) mutant, this results in 1,556 high confidence differentially spliced transcripts. D1 indicates neuronal differentiation set number. C. A graph of Gene Ontology (GO) term enrichment of 1,556 differentially spliced transcripts in *SNCA* (A30P) mutant protein-expressing DA neuronal cells compared to the edited wild type control (EWT). These genes are involved in regulation of cell projection organization, regulation of neuron differentiation and regulation of GTPase activity. D. Comparison of differentially spliced transcripts in *SNCA* (A30P) mutant cells to differentially spliced transcripts from genes found in Lewy body disease patient brain biopsy RNA-seq data (Feleke et al., 2021): (Parkinson disease (PD), Parkinson disease with dementia (PDD), dementia with Lewy bodies (DLB)). E. A volcano plot of D1 (differentiation set 1) is shown as an example with some up- and down-regulated gene names shown. DESeq2 analysis of the *SNCA* (A30P) mutant compared to the edited wild type (EWT) controls reveals 2,338 differentially expressed genes (adjusted p-values < 0.05, FC > 1.5). F. A graph of Gene Ontology (GO) term enrichment of either 1,394 up-regulated genes or 944 down-regulated genes in the *SNCA* (A30P) mutant protein-expressing mDA neuronal cells compared to the edited wild type (EWT) controls.

Alpha-synuclein (SNCA) is a prominent component in Lewy bodies and aggregation of this protein is commonly used as a marker for PD (Kruger et al., 1998). For the *SNCA* A30P mutation, a significant number of splicing pattern changes were observed compared to the EWT controls with 1556 high confidence altered transcripts from two independent A30P mutant cell clones (Fig. 3A and 3B). Here, this group of pre-mRNAs are linked to GO terms for cytoskeletal organization, cell projection organization and regulation of GTPase activity (Fig. 3C). These genes are interesting given the defects in cilia, vesicle trafficking and the involvement of Rab proteins in PD (Bellucci et al., 2022, Bonet-Ponce and Cookson, 2019, Tian et al., 2024). Again, there is overlap in splicing changes between the *SNCA* A30P mutant hPSC-derived neurons and many of the transcript isoforms altered in all three Lewy body diseases (Fig. 3D). The genes whose transcripts are affected at the level of pre-mRNA splicing are indicated in Supplemental Tables S1 and S2. Differential expression profiling of transcripts from *SNCA* A30P mutant cells revealed genes that are involved in synaptic signaling and regulation of exocytosis are up-regulated in *SNCA* A30P mutants while genes are involved in cell adhesion, cell differentiation and cell motility are down-regulated in these mutants compared to the EWT controls (Fig. 3E and 3F). Differentially expressed genes from the GO enrichment analysis are listed in Supplemental Table S3.

### SNCA A53T

**Figure 4.**
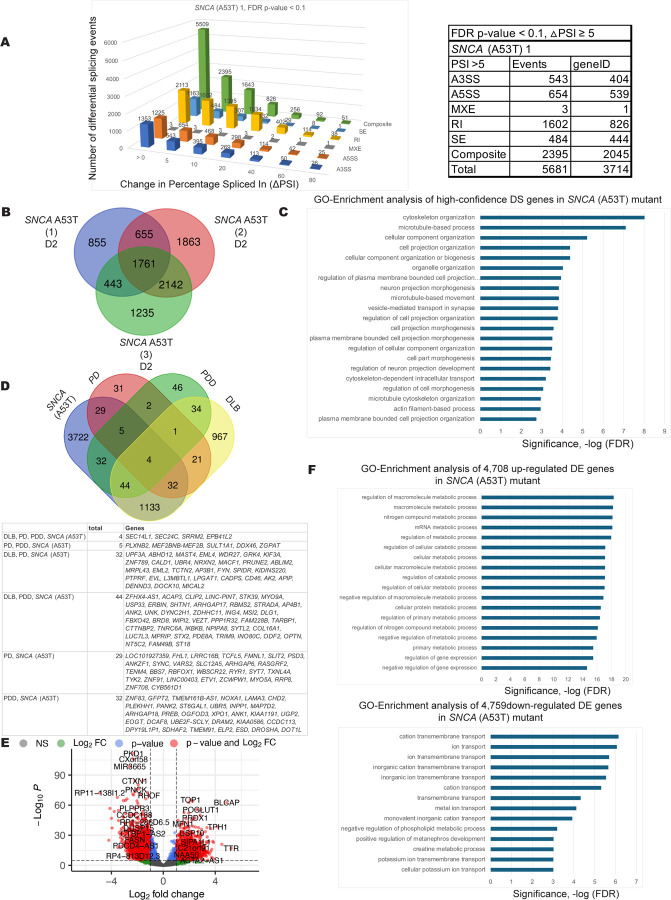
Differentially spliced and differentially expressed transcripts in DA neuronal cells expressing the *SNCA* (A53T) mutation. A. Differential AS events detected upon *SNCA* (A53T) mutation in differentiated DA neurons and progenitor cells (35d) using JUM (Wang and Rio, 2018). 5,001 splicing events were significantly altered in *SNCA* (A53T) mutant samples in DA neurons and progenitor cell line versus the edited wildtype control (with adjusted p-value < 0.1, ΔPSI ≥5). Data for one of three clonal mutant cell lines is shown as an example. Shown on the y-axis is the number of differential splicing events and difference in percentage-spliced-in (or ΔPSI) is shown on the x-axis. The splicing events were filtered based on the magnitude of delta PSI (ΔPSI). B. Comparison of genes that are differentially spliced in 3 different clonal lines of *SNCA* (A53T) mutant, this results in 5,001 high confidence differentially spliced genes. D2 indicates neuronal differentiation sets. C. A graph of Gene Ontology (GO) term enrichment of 5,001 differentially spliced transcripts in *SNCA* (A53T) mutant protein-expressing DA neuronal cells compared to the edited wild type control (EWT). D. Comparison of differentially spliced transcripts in *SNCA* (A53T) mutant cells to differentially spliced transcripts from genes found in Lewy body disease patient brain biopsy RNA-seq data (Feleke et al., 2021): (Parkinson disease (PD), Parkinson disease with dementia (PDD), dementia with Lewy bodies (DLB)). E. A volcano plot of D2 (differentiation set 2) is shown as an example with some up- and down-regulated gene names shown. DESeq2 analysis of the *SNCA* (A53T) mutant compared to the edited wild type (EWT) controls reveals 9,467 differentially expressed genes (adjusted p-values < 0.05, FC > 1.5). F. A graph of Gene Ontology (GO) term enrichment of either 4,708 up-regulated genes or 4,759 down-regulated genes in the *SNCA* (A53T) mutant protein-expressing mDA neuronal cells compared to the edited wild type (EWT) controls.

There are multiple mutations and genetic variations in the alpha-synuclein (SNCA) gene that drive Parkinson's disease (PD) pathology. While the A53T mutation is a well-known example, others include the previously mentioned A30P mutation and the E46K mutation, as well as gene duplications and triplications (Polymeropoulos et al., 1997, Zarranz et al., 2004, Kruger et al., 1998, Singleton et al., 2003, Chartier-Harlin et al., 2004). For the *SNCA* A53T mutation, splicing pattern changes were observed compared to the EWT controls with 5001 high confidence transcripts from three independent A53T mutant cell clones (Fig. 4A and 4B). This group of pre-mRNAs are linked to GO terms for cytoskeletal organization, cell projection organization and intracellular transport (Fig. 4C). These genes are interesting given the defects in cilia and vesicle trafficking linked to PD (Tian et al., 2024, Komori and Kuwahara, 2023, Pfeffer, 2023). Notably, there is overlap in splicing changes between the *SNCA* A53T mutant hPSC-derived neurons and many of the transcript isoforms altered in all three Lewy body diseases (Fig. 4D). The genes whose transcripts are affected at the level of pre-mRNA splicing are indicated in Supplemental Tables S1 and S2. DESeq2 expression level analysis revealed genes involved in metabolic processes were up-regulated while genes involved in ion transport were down-regulated in the *SNCA* A53T mutant cells (Fig. 4E and 4F). Differentially expressed genes from the GO enrichment analysis are listed in Supplemental Table S3.

### LRRK2 G2019S

**Figure 5.**
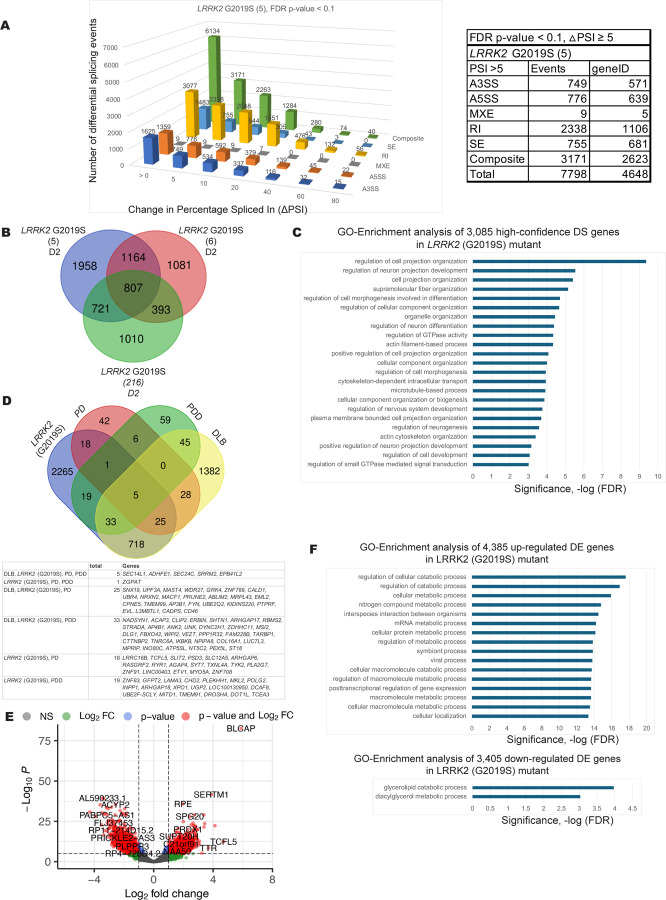
Differentially spliced and differentially expressed transcripts in DA neuronal cells carrying the familial *LRRK2 (G201*9S) mutation. A. Differential alternative splicing events detected upon *LRRK2* (G2019S) in differentiated DA neurons and progenitor cells (35d) using JUM (Wang and Rio, 2018). 3,085 splicing events were significantly altered in *LRRK2* (G2019S) mutant samples in DA neurons and progenitor cell line versus the edited wild type control (with adjusted p-value < 0.1, ΔPSI ≥5). Data for one of three clonal mutant cell lines is shown as an example. Shown on the y-axis is the number of differential splicing events and difference in percentage-spliced-in (or ΔPSI) shown on the x-axis. The splicing events were filtered based on the magnitude of delta PSI (ΔPSI). B. Comparison of gene transcripts that are differentially spliced in three independent clonal lines of the *LRRK2* (G2019S) mutant resulted in 3,085 high confidence differentially spliced genes. D2 indicates neuronal differentiation set. C. A graph of Gene Ontology (GO) term enrichment of 3,085 differentially spliced transcripts in *LRRK2* (G2019S) mutant protein-expressing DA neuronal cells compared to the edited wild type control (EWT). These genes are involved in regulation of cytoskeleton organization, regulation of neuron differentiation, and cilium organization. D. Comparison of differentially spliced transcripts in the *LRRK2* (G2019S) mutant cells to differentially spliced transcripts from genes found in Lewy body disease patient brain biopsy RNA-seq data (Feleke et al., 2021): (Parkinson disease (PD), Parkinson disease with dementia (PDD), dementia with Lewy bodies (DLB)). E. A volcano plot of D1 (differentiation set 2) is shown as an example with some up- and down-regulated gene names shown. DESeq2 analysis of the *LRRK2* (G2019S) mutant compared to the edited wild type (EWT) controls reveals 7,790 differentially expressed genes (adjusted p-values < 0.05, FC > 1.5). F. A graph of Gene Ontology (GO) term enrichment of either 4685 up-regulated genes or 3405 down-regulated genes in the *LRRK2* (G2019S) mutant protein-expressing mDA neuronal cells compared to the edited wild type (EWT) controls.

The mutations in *LRRK2* that are associated with aberrant kinase activity is a common and important inherited mutation linked to PD, where a series of kinase inhibitor molecules have been identified (Lesage and Brice, 2009, Taymans and Greggio, 2016). The G2019S mutation leads to a hyperactive form of the kinase (Greggio and Cookson, 2009, Jaleel et al., 2007, West et al., 2007). In the mutant hPSC-derived mDAs carrying the G20191S mutation there are 3085 high confidence RNA splicing changes derived from three cell clones that were found in two of the three mutant cell lines (Fig. 5A and 5B). The genes whose transcripts are affected are linked to the cytoskeleton, GTPase regulation and microtubule-based processes (Fig. 5C). Many of these altered pre-mRNA splicing changes are also observed in Lewy body patient biopsy RNA-seq data sets (Fig. 5D). The genes whose transcripts are affected at the level of pre-mRNA splicing are indicated in Supplemental Tables S1 and S2. Differential expression profiling analysis indicated genes involved in metabolic processes and posttranscription regulation of gene expression are up-regulated in the *LRRK2* G2019S mutant, while genes involved in glycerolipid catabolic processes are down-regulated (Fig. 5E and 5F). Supplemental Table S3 contains the list of differentially expressed genes resulting from the GO enrichment analysis.

### PINK1 Q129X1

PINK1 is a protein kinase involved in mitochondrial quality control. The *PINK1* Q129X1 mutation is genetically linked to PD (Valente et al., 2004, Beilina et al., 2005, Ibanez et al., 2006, Ishihara-Paul et al., 2008). For this mutation, splicing pattern changes were observed compared to the EWT controls with 2905 high confidence transcripts from three independent *PINK1* mutant cell clones (Supplemental Fig. S1A and S1B). This group of pre-mRNAs are linked to GO terms for cell projection organization, neuronal differentiation and vesicle transport in neurons (Supplemental Fig. S1C). Again, these genes are interesting given the defects in cilia and vesicle-mediated transport linked to PD (Pfeffer, 2023, Komori and Kuwahara, 2023, Tian et al., 2024). There is also overlap in splicing changes between the *PINK1* mutant hPSC-derived mDA neurons and many of the transcript isoforms altered in all three Lewy body diseases (Supplemental Fig. S1D). The genes whose transcripts are affected at the level of pre-mRNA splicing are indicated in Supplemental Tables S1 and S2. Differential expression profiling analysis revealed *PINK1-AS1* anti-sense RNA is predominantly down-regulated in *PINK1* Q129X1 mutant cells compared to the EWT controls (Supplemental Fig. S1E and S1F). It will be interesting to investigate PD patients with the *PINK1* Q129X1 mutation to study if there is an altered expression or regulation of the *PINK1-AS1* non-coding RNA specifically in these PD patients. A list of the differentially expressed genes associated with the GO enrichment terms can be found in Supplemental Table S3.

### SYNJ1 R258Q

SYNJ1 is a lipid phosphatase involved in endosomal trafficking and vesicle recycling and mutations in *SYNJ1* have been linked to PD and several neurological disorders (Choudhry et al., 2021, Drouet and Lesage, 2014, Soukup et al., 2018). The *SYNJ1* R235Q mutation leads to 1954 high confidence splicing changes compared to the EWT controls (Supplemental Fig. S2A and S2B). The genes whose pre-mRNA splicing patterns are altered in *SYNJ1* mutant differentiated cells are linked to cytoskeletal organization, microtubule-based processes and cilium organization (Supplemental Fig. S2C). Splicing changes observed in the *SYNJ1* mutant differentiated hPSCs also overlap with many altered transcript isoforms found in Lewy body disease patient brain samples (Supplemental Fig. S2D). The genes whose transcripts are affected at the level of pre-mRNA splicing are indicated in Supplemental Tables S1 and S2. DESeq2 analysis revealed that genes involved in mRNA metabolic processes and regulation of gene expression are up-regulated in *SYNJ1* R258Q, while genes related to ion transport were down-regulated in the *SYNJ1* R258Q mutant compared to the EWT controls (Supplemental Fig. S2D and S2F). Differentially expressed genes from the GO enrichment analysis are listed in Supplemental Table S3.

### FBXO7 frameshift (FS)

FBXO7 is a ubiquitin ligase that regulates the cell cycle and mediates mitochondrial clearance and mutations in this gene have been linked to PD (Di Fonzo et al., 2009, Zhou et al., 2018). The *FBXO7* frameshift mutation leads to 4752 splicing changes compared to EWT controls (Supplemental Fig. S3A). In this case, only 1 mutant cell line passed the quality control metrics. The genes whose splicing patterns are altered in *FBXO7* mutant cells are linked to regulation of GTPase activity, cytoskeletal organization and cilium assembly (Supplemental Fig. S3B). The changes in pre-mRNA splicing observed in mDA cells with the *FBXO7* mutation were similar to many of the altered transcript isoforms found in brain tissue from patients with Lewy body disease. This suggests that the same splicing defects caused by the *FBXO7* mutation may also contribute to the pathology seen in Lewy body diseases (Supplemental Fig. S3C). The genes whose transcripts are affected at the level of pre-mRNA splicing are indicated in Supplemental Tables S1 and S2. Analysis of gene expression levels indicated that genes involved in protein targeting to the endoplasmic reticulum (ER) and establishment of protein localization to the ER are up-regulated in the *FBXO7* FS mutant while genes involved in the semaphorin-plexin pathway involved in axon guidance and regulation of AMPA receptor activities are downregulated (Supplemental Fig. S3D and S3E). The differentially expressed genes identified through GO enrichment analysis are detailed in Supplemental Table S3.

### DNAJC6 Frameshift (FS)

DNAJC6 is a co-chaperone protein implicated in vesicle transport and lipid homeostasis. It is found mutated in early onset PD patients (Edvardson et al., 2012, Olgiati et al., 2016). The *DNAJC6* frameshift mutation resulted in 4933 high-confidence pre-mRNA splicing changes in the bulk differentiated hPSCs from two mutant cell line clones (Supplemental Fig. S4A and S4B). Genes whose transcript splicing patterns were altered are linked to cell projection organization, GTPase activity regulation, vesicle trafficking and cilium assembly (Supplemental Fig. S4C). There is significant overlap in the pre-mRNA splicing isoforms that are altered in the *DNAJC6* mutant cell line and those found in RNA-seq data from Lewy body disease patient biopsies (Supplemental Fig. S4D). The genes whose transcripts are affected at the level of pre-mRNA splicing are indicated in Supplemental Tables S1 and S2. DESeq2 expression analysis revealed genes involved in co-translational protein targeting to membranes and protein targeting to the ER are up-regulated, while genes involved in regulation of trans-synaptic signaling and neuron projection morphogenesis are down-regulated (Supplemental Fig. S4E and S4F). One noteworthy observation is that the *FOXA2* gene is significantly down-regulated in the *DNAJC6* FS mutant (Figure 1B). Since *FOXA2* plays a role in the survival and function of dopamine neurons (Kittappa et al., 2007, Oh et al., 2016, Domanskyi et al., 2014) the reduced expression or function of *FOXA2* gene in the *DNAJC6* FS mutant may contribute to the neurodegeneration in Parkinson's disease. Differentially expressed genes from the GO enrichment analysis are listed in Supplemental Table S3.

### DJ1 *X1–5DEL*

DJ1 is critical for mitochondrial function and mitophagy (Canet-Aviles et al., 2004, Thomas et al., 2011, Dodson and Guo, 2007). Loss-of-function mutations impair ATP production and exacerbate oxidative stress via dysfunctional mitochondrial accumulation and reduced antioxidant capacity, contributing to neurodegeneration in Parkinson's disease. DJ1, encoded by the *PARK7* gene, is a protein that protects cells from oxidative stress and mutations in the *PARK7* gene have been linked to early onset PD (Bonifati et al., 2003b, Bonifati et al., 2003a, Pankratz et al., 2006). Our analysis of pre-mRNA splicing pattern changes from differentiated hPSCs carrying the DJ1 X1-5 deletion (DJ1 X1-5DEL) exhibited 6625 high-confidence pre-mRNA splicing pattern changes from three mutant cell clones (Supplemental Fig. S5A and S5B). The genes whose pre-mRNA splicing patterns were altered were linked by GO terms to cilium assembly/organization and cell projection organization (Supplemental Fig. 5C). A significant fraction of transcripts affected in the DJ1 mutant cell line are also found to be altered in the RNA-seq data from Lewy body disease patients (Supplemental Fig. S5D). The genes whose transcripts are affected at the level of pre-mRNA splicing are indicated in Supplemental Tables S1 and S2. Differential expression analysis revealed genes involved in co-translational protein targeting to membranes and protein targeting to the ER are upregulated in the DJ1 X1-5DEL mutant, while genes involved in RNA splicing are down-regulated in this mutant compared to the EWT controls (Supplemental Fig. S5E and S5F). Several mitochondrial genes are also downregulated (Supplemental Fig. S5E). A list of the differentially expressed genes associated with the GO term enrichment can be found in Supplemental Table S3.

### VPS13C W395C

The *VPS13C* gene is a gene involved in vesicle sorting, mitochondrial-lysosome function and lipid transfer between membranes. Recessive mutations in this gene are linked to early onset PD (Schormair et al., 2018, Smolders et al., 2021, Lesage et al., 2016). Our analysis of the *VPS13C* W395C mutation identified 4175 pre-mRNA splicing changes from two differentiated mutant cell clones (Supplemental Fig. S6A and S6B). The genes whose pre-mRNA isoforms were altered in the *VPS13C* mutant cells were linked to metabolic processes, chromatin modification and RNA splicing (Supplemental Fig. S6C). There could be a connection between mitochondrial function and RNA splicing since the process uses ATP at multiple steps. Comparison of the splicing events affected in *VPS13C* mutant differentiated hPSCs and the RNA-seq data from Lewy body patient brains indicates overlap between the transcripts whose splicing is affected, with DLB having the most overlap (Supplemental Fig. S6D). The genes whose transcripts are affected at the level of pre-mRNA splicing are indicated in Supplemental Tables S1 and S2. DESeq2 analysis revealed differential gene expression profiles for the up-regulation of genes involved in cell adhesion and down-regulation of genes involved in splicing and regulation of gene expression. Down-regulation of mitochondrial genes is also predominant in the *VPS13C* W395C mutant (Supplemental Fig. S6E and S6F). Some studies suggest *VPS13C* plays a role in mitophagy (Lesage et al., 2016, Wang et al., 2025, Schrӧder et al., 2024). Loss of *VPS13C* could impair mitochondrial homeostasis due to compromised lysosomes, leading to the accumulation of dysfunctional mitochondria and increased oxidative stress. Differentially expressed genes from the GO enrichment analysis are listed in Supplemental Table S3.

### GBA1 IVS2

The *GBA1* gene encodes a lysosomal enzyme that maintains glycosphingolipid homeostasis (Huh et al., 2023, Sidransky et al., 2009). Mutations in the GBA1 gene, such as the c.115+1G>A variant, are among the most common risk factors found in PD patients, as they can disrupt the gene's splicing pattern, leading to a non-functional glucocerebrosidase protein (Onal et al., 2024, Neumann et al., 2009). Using mDA carrying a common mutation in *GBA1* intron 2 (a genotype typically associated with Gaucher disease, not the more common heterozygous GBA1 mutations seen in Parkinson's disease), we found 1857 high-confidence pre-mRNA splicing changes from two mutant cell line clones (Supplemental Fig. S7A and S7B). Genes whose splicing was affected include cell projection, cytoskeletal organization and regulation of GTPase activity (Supplemental Fig. S7C). There is overlap between the splicing event changes detected in the *GBA1* mutant cell line and the altered transcript isoforms from Lewy body disease brain biopsy RNA-seq data (Supplemental Fig. S7D). The genes whose transcripts are affected at the level of pre-mRNA splicing are indicated in Supplemental Tables S1 and S2. Expression profiling analysis indicated genes involved in mitotic cell cycle checkpoint and microtubule-based processes are up-regulated, while genes involved in regulation of trans-synaptic signaling and regulation of transport are down-regulated in the *GBA1* IVS2 mutant compared to the EWT controls (Supplemental Fig. S7E and S7F). The differentially expressed genes identified through GO enrichment analysis are detailed in Supplemental Table S3.

### ATP13A2 Frameshift (FS)

The *ATP13A2* (*PARK9*) gene encodes a lysosomal ion transport protein that functions in acidification of lysosomes. Mutations in the *ATP13A2* gene are linked to Kufor-Rakeb Syndrome, a form of early onset PD (Park et al., 2015, Ramirez et al., 2006, Fujii et al., 2023). The analysis of the altered pre-mRNA splicing patterns in mDA carrying a frameshift mutation in the *ATP13A2* gene indicates that there are 1181 high-confidence splicing changes from two mutant cell line clones compared to the EWT controls (Supplemental Fig. S8A and S8B). The genes whose transcript isoforms were altered include some linked to the cytoskeleton, actin filaments and regulation of GTPase activity (Supplemental Fig. S8C). There is overlap between the splicing event changes detected in the *ATP13A2* mutant cell line and the altered transcript isoforms from Lewy body disease brain biopsy RNA-seq data (Supplemental Fig. S8D). The genes whose transcripts are affected at the level of pre-mRNA splicing are indicated in Supplemental Tables S1 and S2. Differential gene expression analysis of the *ATP13A2* FS mutant revealed that genes involved in modulation of chemical synaptic transmission and transmembrane transport are up-regulated, while genes involved in extracellular matrix organization were down-regulated compared to the EWT controls (Supplemental Fig. S8E and S8F), including the *ATP13A2* gene itself (Supplemental Table S6). Studies have reported decreased ATP13A2 protein levels in the substantia nigra dopaminergic neurons and frontal cortex of PD patients compared to controls (Fujii et al., 2023, Kitada et al., 1998, Dehay et al., 2012). Differentially expressed genes associated with the GO term enrichment can be found in Supplemental Table S3.

### Overlap of GO Enrichment in Differential Splicing and Expression Profiling

We repeatedly observed several GO-term enrichment terms in our differential splicing and differential expression profiling analysis in the familial PD mutants (Supplemental Tables S2 and S3). These GO terms for differentially spliced transcripts include regulation of cell projection organization, cytoskeleton organization, regulation of GTPase activity, cilium organization and cilium assembly. Disruptions in cellular architecture and trafficking mechanisms are increasingly implicated in PD pathogenesis. The organization of neuronal projections, particularly axons, relies on a dynamic cytoskeleton (Guo et al., 2020) and evidence suggests that alpha-synuclein accumulation, a hallmark of PD, directly impairs microtubule stability and axonal transport (Pellegrini et al., 2017, Carnwath et al., 2018). Furthermore, the PD-associated kinase LRRK2 regulates Rab GTPase activity, crucial for vesicular trafficking, and pathogenic *LRRK2* mutations can lead to aberrant Rab phosphorylation, disrupting essential cellular transport pathways (Boecker, 2023, Pfeffer, 2023, Taylor and Alessi, 2020). LRRK2 has been shown to influence ciliogenesis and *LRRK2* mutations correlate with cilia loss in vulnerable neuronal populations (Berwick et al., 2021, Alessi and Pfeffer, 2024). This loss of cilia and subsequent disruption of Shh signaling may compromise the survival of dopaminergic neurons. Indeed, reduced cilia prevalence has been observed in postmortem PD brains (Tian et al., 2024).

### Overlap of differential pre-mRNA transcript spliced isoform expression between PD patient brain (anterior cingulate cortex) postmortem data with splicing alterations from hPSC-derived mDA cells carrying familial PD mutations.

We used a publicly available RNA-sequencing dataset from a study by Feleke et al. (2021) and reanalyzed it with our own software, JUM, for differential splicing analysis. The dataset includes postmortem brain cortex samples from 19 patients with different Lewy body diseases and 5 healthy controls, which we used for comparison with our own hPSC data. The Ryten group used Leafcutter (Li et al., 2018) for alternative splicing pattern analysis and determined that 305 PD-associated genes had splicing pattern changes (Feleke et al., 2021). Leafcutter uses an intron-centric view of splicing and therefore all detected splicing events are given as intron coordinates in split-read clusters. It defines AS events as clusters of overlapping introns which may involve multiple alternative 3’ and 5’ splice sites and skipped exons, making it hard to determine the actual patterns and modes of alternative splicing used by specific splicing junctions. Hence, we took the Ryten lab’s raw data (which was deeply sequenced at 150–250 million PE150 Illumina reads) and analyzed the data using JUM (Wang and Rio, 2018), which allowed us to determine AS modes and patterns, while yielding a quantitative comparison of PSI values for usage of all detected splice junctions.

We compared the postmortem brain cortex data analyzed by either JUM or Leafcutter (Supplemental Table S9) to the RNA-seq data from the hPSC-derived mutant mDA neuron cultures (Fig. 2D–5D and Supplemental Fig. S1D-S8D; Supplemental Fig. S9). Because JUM and Leafcutter use different algorithms for splicing pattern analysis, as well as different cutoffs for statistical analysis, there was some overlap but also differences in terms of the number of differentially spliced transcripts between the software programs. While there were slightly fewer splicing changes found by JUM in the PD patient data, the general trend was the same, in which the most differential splicing events identified in our cell models were observed in patients with DLB compared to PD or PDD (Fig. 2D–5D and Supplemental Fig. S1D-S8D; Supplemental Fig. S9). This analysis shows that alternative splicing is an important mechanism in the development of Lewy body diseases, including PD and can be used to distinguish these three clinically distinct Lewy body diseases (Supplemental Fig. S9).

**Figure 6.**
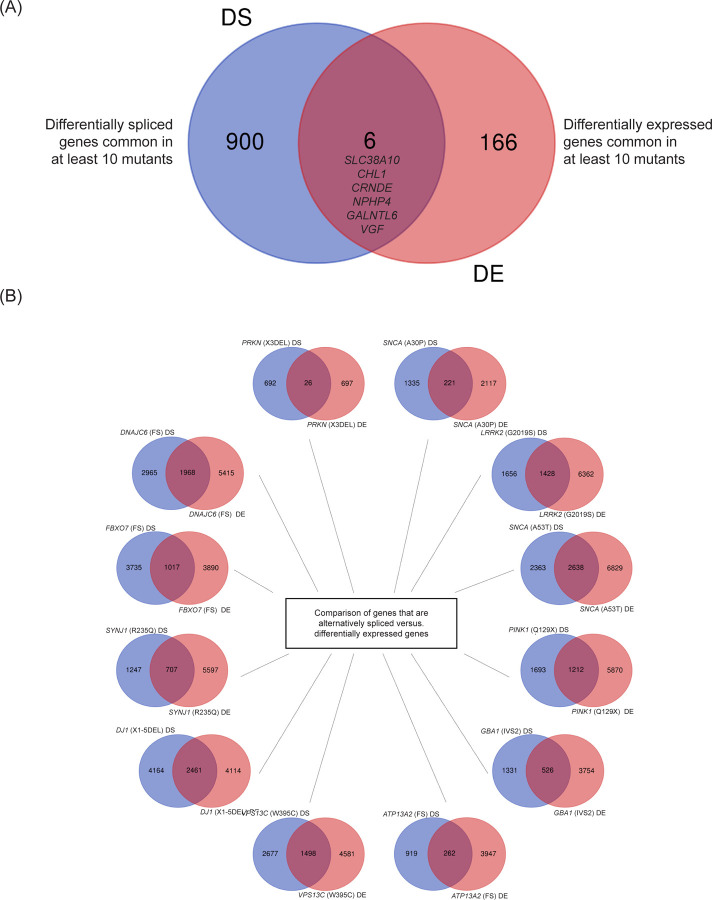
Alternative splicing and gene expression are subject to distinct and generally non-overlapping regulation. (A) Comparison of differentially spliced (DS) versus differentially expressed (DE) gene transcripts that are common in at least 10 familial PD-associated mutants. 906 genes with significant splicing alterations were detected in 10 more distinct familial PD mutants, while 172 genes with altered expression patterns are detected in common. (B) Venn diagrams showing overlap between genes whose transcripts are differentially spliced (DS) compared to differentially expressed (DE) in familial PD-associated mutant differentiated mDA neurons. A substantial number of splicing variations are observed without corresponding changes in overall gene expression at the transcript level.

We next sought to pinpoint genes commonly affected by familial PD-associated mutations, focusing on those showing differential splicing or expression profiles. This approach identified 906 genes with significant splicing alterations and 172 genes with altered expression patterns (Fig. 6A). Comparative analysis of these two gene sets revealed a core group of six genes - *SLC38A10*, *CHL1*, *CRNDE*, *NPHP4*, *GALNTL6*, and *VGF* displayed changes in both splicing and expression across a broad spectrum of familial PD mutations. These genes participate in diverse neuronal processes relevant to PD: *SLC38A10* in glutamine metabolism, *CHL1* in dopamine neuron development, *NPHP4* in cilia function, and *VGF* as a potential PD biomarker. While the direct role of some of these genes in PD requires further investigation, future research into their involvement is warranted. Overall, it is apparent that in many cases there is many non-overlapping changes in splicing versus gene expression in these datasets indicating that changes in protein isoforms due to alternative splicing would not be apparent from gene expression analysis (Fig. 6B).

**Figure 7.**
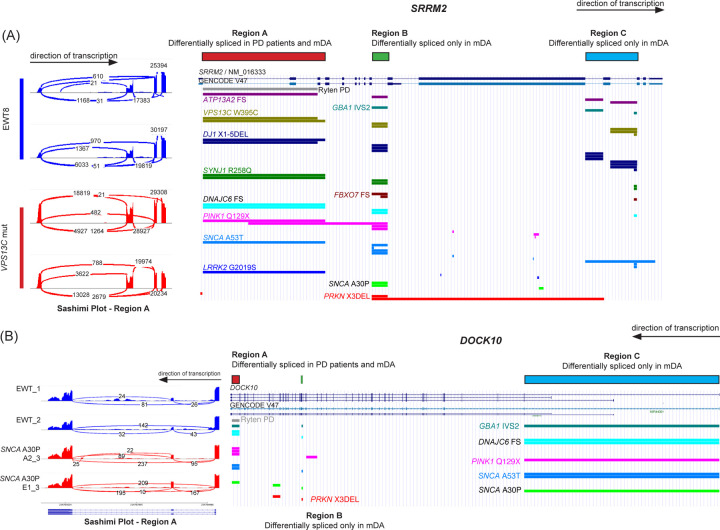
The transcripts from the *SRRM2* and *DOCK10* genes are differentially spliced in midbrain DA neuronal cells expressing the familial PD mutations and in PD patient brain biopsies. Schematic genome browser diagrams with exons indicated at the top and the familial mutants indicated. Sashimi splicing pattern plots of the differentially spliced regions from the *SRRM2* (region A shown in 7A) and *DOCK10* (region A shown in 7B) genes are shown with the number of splice junction reads indicated for the mutant and EWT control for each splicing event (at left, sashimi plot). The two areas indicated Region A show splicing pattern changes for 10 (*SRRM2*) or 5 (*DOCK10*) of the hPSC-derived familial PD mutant DA neuronal data and in the Lewy body disease patient data (Feleke et al., 2021).

Overall, comparing the splicing pattern changes identified by JUM in 12 PD mutant cell lines to those previously reported by the Ryten group (Feleke et al., 2021), we found that there were common genes whose transcript isoforms changed and overlapped between many of the mutant cell lines and the PD patient brain sample datasets (Fig. 2D–5D and Supplemental Fig. S1D-S8D). Interestingly, these included the splicing factor *SRRM2* and the guanine nucleotide exchange factor *DOCK10*, whose transcript splicing patterns were altered in both the PD patient brain samples and the hPSC-derived mDA cells (Fig. 7A and 7B). We analyze both *SRRM2* and *DOCK10* in more detail below.

### Transcripts from the pre-mRNA splicing factor SRRM2 and the Rho family guanine nucleotide exchange factor DOCK10 are differentially spliced in a majority of the familial PD mutant mDA cells.

To identify splicing changes with potential broad functional relevance in PD, we prioritized genes whose transcript splicing patterns were consistently altered across the majority of mutant mDA cells and overlapped with those observed in PD patient brain biopsy data. We found two genes to examine in more detail, the splicing factor *SRRM2* and the guanine nucleotide exchange factor *DOCK10. SRRM2* is a well-known pre-mRNA splicing factor, and consistent with our findings, splicing dysregulation of the RNA splicing factor *SRRM2* was previously observed in PD patients using microarrays (Shehadeh et al., 2010, Fu et al., 2013). More recently *SRRM2* has been linked to neurodevelopmental disorders (Regan-Fendt et al., 2023) and inflammation (Cui et al., 2023). For *SRRM2*, we found that exon 2, which encodes a glycine, proline and highly charged domain is more included in multiple familial PD mutant cell lines, with the *VPS13C* (W395C) mutant shown as an example (Fig. 7A).

The *DOCK10* gene encodes a protein known to act as a guanine nucleotide exchange factor (GEF) for the CDC42 and Rac1-Rho-family GTPases (Boland et al., 2023). This protein is highly expressed the brain and its depletion results in dendritic spine defects (Jaudon et al., 2015). The DOCK10 protein controls the activity of the CDC42 and Rac1 GTPases which affect the actin cytoskeleton, neuronal survival and connectivity (Stankiewicz and Linseman, 2014). In the example shown for the *SNCA* A30P mutant, there is elevated exon inclusion of a negatively charged amino acid sequence, as well as a minor cryptic exon that inserts a stop codon which could lower protein levels (Fig. 7B).

Our analyses indicate that the pre-mRNA splicing pattern changes seen in the hPSC-derived PD familial mutant cells partially overlap and are often the same as some of those found in the PD patient postpmortem RNA-seq data sets (Fig. 7A and 7B, Fig. 2D–5D and Supplemental Fig. S1D to S8D). These RNA splicing alterations could affect other RNA splicing events controlled by *SRRM2* or control cell survival or connections by affecting *DOCK10* activity. Indeed, *Rac1* (Ras-related C3 botulinum toxin substrate 1) has been implicated in PD (Stankiewicz and Linseman, 2014). *SRRM2* is known to be highly expressed in the brain and control RNA splicing patterns of multiple pre-mRNAs linked to autism (Engal et al., 2024). Loss of function mutations in the *SRRM2* gene cause neurodevelopmental disorders (Cuinat et al., 2022).

We validated several of the detected differential splicing events by reverse transcription-polymerase chain reaction (RT-PCR) (Supplemental Fig. S10). Regions from the *UBR4*, *DOCK3*, and *CAMPTA2* genes are shown as examples from the *GBA1* (FS) mutant and EWT control cells. Ubiquitin protein ligase E3 component n-recognin 4 (*UBR4*) is an E3 ubiquitin-protein ligase that is part of a large protein complex, Silencing Factor of the Integrated Stress Response (SIFI) machinery. It has been shown *SIFI* acts as a stress response silencer, targeting unimported mitochondrial proteins and stress response components for proteasomal degradation (Haakonsen et al., 2024). *UBR4* is differentially spliced in *GBA1* (FS) mutant-expressing cells compared to EWT controls (Supplemental Fig. S10, panel A), which could alter the proteasomal degradation process in *GBA1* (FS) mutant cells. We also observed differential exon usage in the *DOCK3* and *CAMPTA2* transcripts in *GBA1* (FS) mutant cells compared to EWT controls (Supplemental Fig. S10, panels A). Dedicator of Cytokinesis 3 (*DOCK3*) promotes neurite and axonal growth and is involved in regulation of skeletal muscle regeneration and attenuation of neural cell death (Namekata et al., 2010, Namekata et al., 2012, Reid et al., 2020, Samani et al., 2023, Namekata et al., 2013). *DOCK3* dysregulation has been shown to be associated with neurodegenerative disorders, including neurodevelopmental disorders with impaired intellectual development, hypotonia, and ataxia (Alexander and Velinov, 2023, Iwata-Otsubo et al., 2018, Wiltrout et al., 2019, Namekata et al., 2010). In addition, numerous DOCK protein-encoding transcripts from other family genes have been differentially spliced in other PD familial mutant-expressing cells (Supplemental Table 1). The CAMTA family of proteins have been shown to control neuronal calmodulin levels (Vuong-Brender et al., 2021), a critical factor for neuronal function and calcium level dysregulation is a critical factor in neurodegenerative diseases. Differential exon usage in mRNAs from both the *DOCK3* and *CAMTA2* genes was observed in *GBA1* (FS) mutant-expressing cells in comparison to EWT controls, possibly mirroring how critical genes might be differentially spliced in familial-PD mutant expressing cells that could lead ultimately to neuronal cell death.

### Variations in differential gene expression profiling in 35 day-differentiated mDA cells with familial PD mutations compared to control edited wild type cells using bulk RNA sequencing.

We also used the RNA-seq data from the *PRKN, SNCA, LRRK2, PINK1, DNAJC6, FBXO7, SYNJ1,* DJ1*, VPS13C, ATP13A2 and GBA1* mutant and edited wild type control cell lines to analyze overall gene expression (RNA transcript levels) using DESeq2 (Love et al., 2014). This analysis showed that there was differential gene expression in the *LRRK2*, *SNCA* (A53T), *PINK1*, DJ1*, VPS13C* and *SYNJ1* mutant cells that led to altered expression of RNA binding proteins and known splicing factors (Fig. 2–5D and F; Supplemental Fig. S1-S8D and F and Supplemental Table 3). In addition, mutations in the *SNCA (*A30P), *PRKN*, *FBXO7*, *ATP13A2*, *PINK1*, *DNAJC6*, DJ1, *VPS13C* and *SYNJ1* altered expression of genes linked to cell communication, synaptic transmission, membrane and ion transport, neuronal projections and protein trafficking to the membrane (Fig. 2–5D and F; Supplemental Fig. S1-S8D and F and Supplemental Table 3). We note that there is some variability in the extent of differentiation of each mutant or wild type cell clone in a given experiment, but these measurements were made in technical triplicate with 1–3 independent cell clones per mutation. We found that there was often variable overlap between gene expression versus pre-mRNA splicing changes in different mutant cells compared to wild type but that many of the splicing changes are distinct from the transcript level gene expression changes (Fig. 6B). This observation may not be surprising because it has been shown that alternative splicing in the nervous system can often result in mRNA levels whose overall expression does not change but alternative splicing generates mRNA isoforms encoding proteins with altered protein-protein interactions (Ellis et al., 2012, Gonatopoulos-Pournatzis and Blencowe, 2020). These alterations in transcript levels or isoform changes could lead to different levels or composition of protein complexes that would alter cellular phenotypes relevant to PD, including the cytoskeleton, GTPase regulation, cilia, neuronal projections, as well as altered splicing factor networks (see Fig. 2–5, and Supplemental Fig. S1-S8; Supplemental Tables 1–3).

Taken together, these experiments provide a comprehensive list of genes whose expression levels change in cells carrying 12 distinct familial PD mutations when the hPSCs are differentiated to mDA neurons *in vitro*. Many of the genes whose expression levels are affected, like Rab family members (Bellucci et al., 2022, Bonet-Ponce and Cookson, 2019, Alessi and Pfeffer, 2024) and SNCA (Singleton et al., 2003, Dauer and Przedborski, 2003, Polymeropoulos et al., 1997) itself are linked to PD, as well as RNA binding proteins, such as TARDBP/TDP-43 and FUS (Mackenzie et al., 2010, Neumann et al., 2006, Hewitt et al., 2010, Moens et al., 2025) which have been linked to ALS (Supplemental Table 3).

## Discussion

Parkinson's disease (PD) lacks a cure, and L-DOPA, its main treatment, loses efficacy and causes side effects over time (Kwon et al., 2022, Dias et al., 2023). Early detection is challenging due to limited biomarkers (Concha-Marambio et al., 2023, Fernandes Gomes et al., 2023). Intensive research is vital to uncover underlying mechanisms for developing early diagnostics and new therapies (Schekman and Riley, 2019). Defects in protein homeostasis, aggregation, RNA binding proteins and RNA processing lead to pathologic physiological changes in PD, as is the case for other neurodegenerative diseases, like amyotrophic lateral sclerosis (ALS) and spinal muscular atrophy (SMA) (Brinegar and Cooper, 2016, Conlon and Manley, 2017, Mann and Donnelly, 2021). Identifying RNA processing events and pre-mRNA splicing isoforms linked to these disease states has led to identification of target disease-relevant transcripts for therapeutic interventions using antisense oligonucleotides (ASOs) (Bennett et al., 2019, Meyer et al., 2020), small molecules (Meyer et al., 2020), and gene therapy (Li et al., 2023). Given the advances in the delivery of nucleic acid therapeutics (Damase et al., 2021) and messenger RNA (Huang et al., 2022), it is likely that once key RNA level targets are identified for PD, therapeutic interventions can be developed. The platform developed and outlined here utilizing hPSCs with PD relevant mutations has the potential to aid in the development of PD therapeutics.

It is clear that for other diseases, such as a variety of cancers, including blood cancers like myelodysplastic syndrome (MDS), and neurodegenerative diseases like SMA, Huntington disease (HD) and ALS, that mutations in RNA binding proteins and known pre-mRNA splicing factors and specific splicing events can play causative roles in the disease states and are now successful therapeutic targets (Gebauer et al., 2021, Kapeli et al., 2017, Rogalska et al., 2023). Many neurodegenerative disease states involve changes in proteostasis, mitochondrial, lysosomal and other metabolic and cell biological pathways (Wilson et al., 2023, Jagtap et al., 2023). In many cases, protein aggregation, for instance the TDP-43 protein in ALS and in PD α-synuclein aggregates are key indicators of disease. In the case of ALS, two key RNA processing events involving the *stathmin 2* (*STMN2*) (Baughn et al., 2023) and *UNC13A* genes (Brown et al., 2022, Ma et al., 2022, Lipstein, 2022) are targets for therapeutic interventions. In addition, splicing alterations in *STMN-2* and *UNC13A* have recently been found in Alzheimer’s disease patients (Agra Almeida Quadros et al., 2024).

Here, we have developed a system to investigate RNA-level changes that are linked to 11 of the 20 most common familial PD hereditary genes, including *PRKN, SNCA, LRRK2, PINK1, DNAJC6, FBXO7, SYNJ1,* DJ1*, VPS13C, ATP13A2 and GBA1* (Poewe et al. 2017). Our results linking splicing pattern alterations to PD are consistent with uncovered defects in global pre-mRNA splicing patterns discovered in other neurodegenerative diseases such as ALS (Li et al., 2021, Wang et al., 2020), SMA (Singh and Singh, 2018) and various cancers (Choi et al., 2023, Zhang and Manley, 2013, Bradley and Anczukow, 2023, Stanley and Abdel-Wahab, 2022, El Marabti and Abdel-Wahab, 2021). It is also clear that mutations in RNA binding protein genes or altered expression levels of RNA binding proteins, such as TDP-43 (Nussbacher et al., 2019, Kapeli et al., 2017) splicing factors and spliceosome components, such as U2AF1 and SF3B1 (Choi et al., 2023, Bradley and Anczukow, 2023, Stanley and Abdel-Wahab, 2022, El Marabti and Abdel-Wahab, 2021) play roles in neurodegeneration and cancer, respectively.

One important finding from this work is that there are many RNA splicing changes, as well as gene expression level changes, in the PD mutant hPSC-derived mDA cells. The PD relevance of these data is supported by analysis of RNA-seq data from PD patient postmortem brain samples (Feleke et al., 2021), which overlaps with some of the splicing pattern changes found in our RNA-seq analysis of hPSC-derived mDA cells carrying familial PD mutations. In our initial studies with the familial *PRKN, SNCA, LRRK2, PINK1, DNAJC6, FBX7, SYNJ1,* DJ1*, VPS13C, ATP13A2 and GBA1* mutants, we have identified splicing pattern changes for the well-known RNA splicing factor *SRRM2* and the guanine nucleotide exchange factor *DOCK10* in all or many of the twelve PD mutant cell lines. These changes in the *SRRM2* mRNA could lead to a cascade of pre-mRNA splicing alterations, since many pre-mRNAs require this factor for correct processing. Interestingly, the *DOCK10* gene regulates the CDC42 and Rac1 GTPases, which are critical for controlling neuronal survival and the actin/microtubule cytoskeletons (Bressan et al., 2023). Thus, the platform we have developed enabled the identification of common defects in RNA processing patterns and RNA splicing factors that occur in mDA cells derived from hPSCs engineered with other PD familial mutations (Busquets et al., 2025).

## STAR ★ METHODS

### CONTACT FOR REAGENT AND RESOURCE SHARING:

Email contact for further information and reagent and resource sharing: don_rio@berkeley.edu and hockemeyer@berkeley.edu.

### METHOD DETAILS

#### Materials availability

All unique/stable reagents generated in this study are available from the Lead Contract without restriction.

### DATA AND SOFTWARE AVAILABILITY

The data reported will be deposited in the Gene Expression Omnibus (GEO) database upon the acceptance of the manuscript.

Publicly available WIBR3 hPSCs RNA-seq data was downloaded (GEO accession numbers: GSM3069316, GSM3069317, GSM3069324, GSM3069325) for comparison to EWT and familial PD mutant cells for expression of neuronal genes in differentiated cells in Figure 1B.

### METHOD DETAILS

#### WIBR3-hPSCs derived DA neuron differentiation:

WIBR3 hPSCs (passage 25–30) were maintained and grown on iMatrix-511 (Takara, T303) coated 6-well plates in mTESR plus media (Stem Cell Technologies, 100–0276). For DA neuron differentiation, the hPSCs were dissociated into single cells and seeded on matrigel (Corning, 356231) coated plates with Rock inhibitor (Chemdea CD0141) at 10 μM to initiate the differentiation. We followed the recently published protocol (Kim et al., 2021) with slight modifications. Briefly, single cell suspension of hPSCs were maintained with Neurobasal N2/B27 (ThermoFisher, 21103049) media containing 2 mM glutamine (Sigma, G8450) with 200 ng/ml SHH C25II (R&D Systems, 1845-SH-100) 250 nM LDN (Stemgent, 04–0074),10 μM SB431542 (SelleckChem, S1067), 0.7 μM CHIR99021 (Tocris, 4423) on day 1 of differentiation. On day 4, CHIR concentration was increased to 7.5 μM and on day 7 LDN, SB, SHH were removed, and media was supplemented with only CHIR. On day 10, media was changed to Neurobasal B27 with 2 mM glutamine supplemented with 20ng/ml BDNF (Peprotech, 450–02), 20 ng/ml GDNF (Peprotech, 450–10), 200 μM ascorbic acid (AA) (Sigma, A40–34) 500 μM dbcAMP (SelleckChem, S7858) and 1 ng/ml TGFb3 (R&D Systems, 8420-B3-005). On Day 11, cells were dissociated with Accutase (ThermoFisher, 00-4555-56) and split into a 1:2 or 1:4 ratio and replated on Poly L-Ornithine (PLO; 30 μg/ml; Sigma, P3655), Cultrex laminin (1 ug/ml; R&D Systems, 3400-010-02) coated plates and maintained in maturation media containing BDNF, GDNF, TGFb3, AA, DAPT from day 12. On day 16, cells were dissociated with Accutase (ThermoFisher, 00-4555-56) and replated at desired high density (1-2x10^6^ cells per well of 12 well plate) depending on cell growth and maintained in maturation medium (at this stage cells were cryopreserved in serum free cryopreservation media cell banker 2 (Amsbio, 11914) and thawed as per the experiment). On day 25, cells were dissociated with Accutase (ThermoFisher, 00-4555-56) and replated at higher density (>1x10^6^ cells per of 12 well plate until the experiment. On day 35, cells were lysed in the RLT lysis buffer using Qiagen kit (RNeasy, 74104) and RNA was isolated for bulk RNA sequencing.

#### RT-PCR validation for splicing analysis

To validate several changes in alternative pre-mRNA splicing patterns found in the RNA-seq data we used reverse-transcription polymerase chain reaction (RT-PCR). In these experiments, we used analysis of *GAPDH* as a loading control. We found alterations in the levels of spliced mRNAs (Supplemental Fig. S8). 0.5 microgram of total RNA was reverse-transcribed according to the manufacturer’s instructions (Bio-Rad, 1708891) and subjected to RT–PCR with the following conditions: 30 sec at 98°C (one cycle); 10 sec at 98°C, 30 sec at 60°C, and 30 sec at 72°C (35 cycles); and 5 min at 72°C (one cycle). The primer sets used in the RT-PCR reaction is listed in Supplement Figure S10B. RT-PCR products were resolved, visualized, and quantitated by use of an 2% agarose gels. Differential splicing events for *UBR4*, *DOCK3*, and *CAMPTA2* were shown as examples in *GBA1* (FS) mut compared to EWT control cells (Supplemental Fig. S10).

#### RNA-seq library preparation and sequencing

RNA isolation was performed using the RNeasy Minikit (Qiagen, 74104), followed by 30 min. of DNase treatment (Ambion, AM2238) at 37°C, poly(A)^+^ RNA transcripts were isolated [NEBNext poly(A) mRNA magnetic isolation module; New England Biolabs, E7490] from 0.5 μg of total RNA for RNA library preparation and sequencing using NEBNext Ultra Directional RNA Library Preparation Kit for Illumina (New England Biolabs, E7420S) according to the manufacturer’s instruction. The samples were sequenced on an Illumina S1 or S4 with 150-bp paired-end reads at the Vincent J. Coates Genomics Sequencing Laboratory at the University of California at Berkeley. Typical samples gave ~200–300M PE150 reads.

#### Analysis of pre-mRNA splicing patterns using JUM and differential expression analysis using DESeq2

Pre-mRNA splicing analysis using JUM.2.0.2 software to detect pre-mRNA splicing patterns was performed as described before (Lee et al., 2018, Wang and Rio, 2018). An adjusted *p*-value (FDR) of 0.1 was used as the statistical cutoff for differentially spliced AS events with at least 10 junction reads.

For Differential expression analysis, uniquely mapped reads were mapped to hg38 using STAR_2.5.3a (Dobin et al., 2013) modified version using --outFilterMultimapNmax 1. Read counts were calculated using HTSeq script htseq-count (Anders et al., 2015) with the following specifications: -s yes -r pos -f. Differential gene expression analysis was performed using DESeq2 (Love et al., 2014), only genes with at least 10 reads across all samples were considered for further analysis.

## Supplementary Material

Supplement 1 **Supplemental Figure S1. Differentially spliced and differentially expressed transcripts in DA neuronal cells expressing the *PINK1* (Q129X) mutation.** A. Differential alternative splicing events detected upon *PINK1* (Q129X) in differentiated DA neurons and progenitor cells (35d) using JUM (Wang and Rio, 2018). 2,905 splicing events were significantly altered in *PINK1* (Q129X) mutant samples in DA neurons and progenitor cell line versus the edited wildtype control (with adjusted p-value < 0.1, ΔPSI ≥5). Data for one of three clonal mutant cell lines is shown as an example. Shown on the y-axis is the number of differential splicing events and difference in percentage-spliced-in (or ΔPSI) shown on the x-axis. The splicing events were filtered based on the magnitude of delta PSI (ΔPSI). B. Comparison of genes that are differentially spliced in 3 different clonal lines of *PINK1* (Q129X) mutant, this results in 2,905 high confidence differentially spliced genes. D2 indicates different neuronal differentiation set number. C. A graph of Gene Ontology (GO) term enrichment of 2,905 differentially spliced transcripts in *PINK1* (Q129X) mutant protein-expressing DA neuronal cells compared to the edited wild type control (EWT). D. Comparison of differentially spliced genes in *PINK1* (Q129X) mutant cells to differentially spliced transcripts from genes found in Lewy body disease patient brain biopsy RNA-seq data (Feleke et al., 2021): (Parkinson disease (PD), Parkinson disease with dementia (PDD), dementia with Lewy bodies (DLB)). E. A volcano plot of D2 (differentiation set 2) is shown as an example with some up- and down-regulated gene names shown. DESeq2 analysis of the *PINK1* (Q129X) mutant compared to the edited wild type (EWT) controls reveals 7,081 differentially expressed genes (adjusted p-values < 0.05, FC > 1.5). F. A graph of Gene Ontology (GO) term enrichment of either 3,998 up-regulated genes or 3,083 down-regulated genes in the *PINK1* (Q129X) mutant protein-expressing mDA neuronal cells compared to the edited wild type (EWT) controls.
**Supplemental Figure S2. Differentially spliced and differentially expressed transcripts in mDA neuronal cells expressing the *SYNJ1* (R235Q) mutation.** A. Differential alternative splicing (AS) events detected in *SYNJ1* (R235Q) mutant differentiated mDA neurons and progenitor cells (35d). Over 1,900 splicing events were significantly altered in *SYNJ1* (R235Q) mutant samples of mDA neurons and progenitor cell lines versus the edited wild type control cells (with adjusted p-value < 0.1, ΔPSI ≥5). Data for one clonal cell line is shown as an example. Shown on the y-axis is the number of differential splicing events and difference in percentage spliced in (or ΔPSI) shown on the x-axis. The splicing events were filtered based on the magnitude of delta PSI (ΔPSI). B. Comparison of transcripts from genes that are differentially spliced in 2 different clonal lines of the *SYNJ1* (R235Q) mutant, which results in 1,954 high confidence differentially spliced gene transcripts. D2 indicates the neuronal differentiation set number. C. A graph of Gene Ontology (GO) term enrichment of 1,954 differentially spliced transcripts in *SYNJ1* (R235Q) mutant protein-expressing mDA neuronal cells compared to the edited wild type control cells. D. Comparison of differentially spliced transcripts in the *SYNJ1* (R235Q) mutant cells to differentially spliced transcripts found in diseased patient brains (Parkinson disease (PD), Parkinson disease with dementia (PDD), dementia with Lewy bodies (DLB)). E. A volcano plot of D2 (differentiation set 2) is shown as an example with some up- and down-regulated gene names shown. DESeq2 analysis of the *SYNJ1* (R258Q) mutant compared to the edited wild type (EWT) controls reveals 6304 differentially expressed genes (adjusted p-values < 0.05, FC > 1.5). F. A graph of Gene Ontology (GO) term enrichment of either 3,211 up-regulated genes or 3,093 down-regulated genes in the *SYNJ1* (R258Q) mutant protein-expressing mDA neuronal cells compared to the edited wild type (EWT) controls.
**Supplemental Figure S3. Differentially spliced and differentially expressed transcripts in mDA neuronal cells expressing the *FBXO7* (FS) mutation.** A. Differential alternative splicing (AS) events detected in *FBXO7* (FS) mutant differentiated mDA neurons and progenitor cells (35d). Over 4,500 splicing events were significantly altered in *FBXO7* (FS) mutant samples of mDA neurons and progenitor cells versus the edited wild type control cells (with adjusted p-value < 0.1, ΔPSI ≥5). Data for one clonal cell line is shown. Shown on the y-axis is the number of differential splicing events and difference in percentage spliced in (or ΔPSI) shown on the x-axis. The splicing events were filtered based on the magnitude of delta PSI (ΔPSI). B. A graph of Gene Ontology (GO) term enrichment of 4,506 differentially spliced transcripts in *FBXO7* (FS) mutant protein-expressing DA neuronal cells against the edited wild type control cells. C. Comparison of differentially spliced transcripts in the *FBXO7* (FS) mutant cell line to differentially spliced transcripts found in diseased patient brains (Parkinson disease (PD), Parkinson disease with dementia (PDD), dementia with Lewy bodies (DLB)). D. A volcano plot of D2 (differentiation set 2) is shown as an example with some up- and down-regulated gene names shown. DESeq2 analysis of the *FBXO7* (FS) mutant compared to the edited wild type (EWT) controls reveals 4,907 differentially expressed genes (adjusted p-values < 0.05, FC > 1.5). E. A graph of Gene Ontology (GO) term enrichment of either 1,841 up-regulated genes or 3,066 down-regulated genes in the *FBXO7* (FS) mutant protein-expressing mDA neuronal cells compared to the edited wild type (EWT) controls.
**Supplemental Figure S4. Differentially spliced and differentially expressed transcripts in mDA neuronal cells expressing the *DNAJC6* (FS) mutation.** A. Differential alternative splicing (AS) events detected in *DNAJC6* (FS) mutant differentiated mDA neurons and progenitor cells (35d). ~5,000 splicing events were significantly altered in *DNAJC6* (FS) mutant samples of mDA neurons and progenitor cells versus the edited wild type control cells (with adjusted p-value < 0.1, ΔPSI ≥5). Data for one clonal line is shown as an example. Shown on the y-axis is the number of differential splicing events and difference in percentage spliced in (or ΔPSI) shown on the x-axis. The splicing events were filtered based on the magnitude of delta PSI (ΔPSI). B. Comparison of transcripts from genes that are differentially spliced in 2 different clonal lines of the *DNAJC6* (FS) mutant, which results in 4,933 high confidence differentially spliced gene transcripts. D2 indicates neuronal differentiation set number. C. A graph of Gene Ontology (GO) term enrichment of 4,933 differentially spliced transcripts in *DNAJC6* (FS) mutant protein-expressing mDA neuronal cells compared to the edited wild type control cells. D. Comparison of differentially spliced transcripts in the *DNAJC6* (FS) mutant cells to differentially spliced transcripts found in diseased patient brains (Parkinson disease (PD), Parkinson disease with dementia (PDD), dementia with Lewy bodies (DLB)). E. A volcano plot of D1 (differentiation set 1) is shown as an example with some up- and down-regulated gene names shown. DESeq2 analysis of the *DNAJC6* (FS) mutant compared to the edited wild type (EWT) controls reveals 7,383 differentially expressed genes (adjusted p-values < 0.05, FC > 1.5). F. A graph of Gene Ontology (GO) term enrichment of either 3,763 up-regulated genes or 3,620 down-regulated genes in the *DNAJC6* (FS) mutant protein-expressing mDA neuronal cells compared to the edited wild type (EWT) controls.
**Supplemental Figure S5. Differentially spliced and differentially expressed transcripts in mDA neuronal cells expressing the DJ1 (X1-5DEL) mutation.** A. Differential alternative splicing (AS) events detected in DJ1 (X1-5DEL) mutant differentiated mDA neurons and progenitor cells (35d). Over 6,500 splicing events were significantly altered in DJ1 (X1-5DEL) mutant samples of mDA neurons and progenitor cells versus the edited wild type control cells (with adjusted p-value < 0.1, ΔPSI ≥5). Data for one clonal line is shown as an example. Shown on the y-axis is the number of differential splicing events and difference in percentage spliced in (or ΔPSI) shown on the x-axis. The splicing events were filtered based on the magnitude of delta PSI (ΔPSI). B. Comparison of transcripts from genes that are differentially spliced in 2 different clonal lines of the DJ1 (X1-5DEL) mutant, which results in 6,625 high confidence differentially spliced gene transcripts. D3 indicates neuronal differentiation set number. C. A graph of Gene Ontology (GO) term enrichment of 6,625 differentially spliced transcripts in DJ1 (X1-5DEL) mutant protein-expressing mDA neuronal cells compared to the edited wild type control cells. D. Comparison of differentially spliced transcripts in the DJ1 (X1-5DEL) mutant cells to differentially spliced transcripts found in diseased patient brains (Parkinson disease (PD), Parkinson disease with dementia (PDD), dementia with Lewy bodies (DLB)). E. A volcano plot of D3 (differentiation set 3) is shown as an example with some up- and down-regulated gene names shown. DESeq2 analysis of the DJ1 (X1-5DEL) mutant compared to the edited wild type (EWT) controls reveals 6,575 differentially expressed genes (adjusted p-values < 0.05, FC > 1.5). F. A graph of Gene Ontology (GO) term enrichment of either 3,594 up-regulated genes or 2,981 down-regulated genes in the DJ1 (X1-5DEL) mutant protein-expressing mDA neuronal cells compared to the edited wild type (EWT) controls.
**Supplemental Figure S6. Differentially spliced and differentially expressed transcripts in mDA neuronal cells expressing the *VPS13C* (W395C) mutation.** A. Differential alternative splicing (AS) events detected in *VPS13C* (W395C) mutant differentiated mDA neurons and progenitor cells (35d). Over 4,000 splicing events were significantly altered in *VPS13C* (W395C) mutant samples of mDA neurons and progenitor cell line versus the edited wildtype control (with adjusted p-value < 0.1, ΔPSI ≥5). Data for one clonal line is shown as an example. Shown on the y-axis is the number of differential splicing events and difference in percentage spliced in (or ΔPSI) shown on the x-axis. The splicing events were filtered based on the magnitude of delta PSI (ΔPSI). B. Comparison of transcripts from genes that are differentially spliced in 2 different clonal lines of *VPS13C* (W395C) mutant, which results in 4,175 high confidence differentially spliced gene transcripts. D3 indicates neuronal differentiation set number. C. A graph of Gene Ontology (GO) term enrichment of 4,175 differentially spliced transcripts in *VPS13C* (W395C) mutant protein-expressing mDA neuronal cells compared to the edited wild type control cells. D. Comparison of differentially spliced transcripts in the *VPS13C* (W395C) mutant cells to differentially spliced transcripts found in diseased patient brains (Parkinson disease (PD), Parkinson disease with dementia (PDD), dementia with Lewy bodies (DLB)). E. A volcano plot of D3 (differentiation set 3) is shown as an example with some up- and down-regulated gene names shown. DESeq2 analysis of the *VPS13C* (W395C) mutant compared to the edited wild type (EWT) controls reveals 6,079 differentially expressed genes (adjusted p-values < 0.05, FC > 1.5). F. A graph of Gene Ontology (GO) term enrichment of either 3,249 up-regulated genes or 2,830 down-regulated genes in the *VPS13C* (W395C) mutant protein-expressing mDA neuronal cells compared to the edited wild type (EWT) controls.
**Supplemental Figure S7. Differentially spliced and differentially expressed transcripts in mDA neuronal cells expressing the *GBA1* (IVS2) mutation.** A. Differential alternative splicing (AS) events detected in *GBA1* (IVS2) mutant differentiated mDA neurons and progenitor cells (35d). Over 1800 splicing events were significantly altered in *GBA1* (IVS2) mutant samples of mDA neurons and progenitor cell line versus the edited wild type control cells (with adjusted p-value < 0.1, ΔPSI ≥5). Data for one clonal line is shown as an example. Shown on the y-axis is the number of differential splicing events and difference in percentage spliced in (or ΔPSI) shown on the x-axis. The splicing events were filtered based on the magnitude of delta PSI (ΔPSI). B. Comparison of transcripts from genes that are differentially spliced in 2 different clonal lines of the *GBA1* (IVS2) mutant, which results in 1,857 high confidence differentially spliced gene transcripts. D4 indicates neuronal differentiation set number. C. A graph of Gene Ontology (GO) term enrichment of 1,857 differentially spliced transcripts in *GBA1* (IVS2) mutant protein-expressing mDA neuronal cells compared to the edited wild type control cells. D. Comparison of differentially spliced transcripts in the *GBA1* (IVS2) mutant cells to differentially spliced transcripts found in diseased patient brains (Parkinson disease (PD), Parkinson disease with dementia (PDD), dementia with Lewy bodies (DLB)). E. A volcano plot of D4 (differentiation set 4) is shown as an example with some up- and down-regulated gene names shown. DESeq2 analysis of the *GBA1* (IVS2) mutant compared to the edited wild type (EWT) controls reveals 4,280 differentially expressed genes (adjusted p-values < 0.05, FC > 1.5). F. A graph of Gene Ontology (GO) term enrichment of either 3,249 up-regulated genes or 2,830 down-regulated genes in the *GBA1* (IVS2) mutant protein-expressing mDA neuronal cells compared to the edited wild type (EWT) controls.
**Supplemental Figure S8. Differentially spliced and differentially expressed transcripts in mDA neuronal cells expressing the *ATP13A2* (FS) mutation.** A. Differential alternative splicing (AS) events detected upon *ATP13A2* (FS) mutant differentiated mDA neurons and progenitor cells (35d). 1,111 splicing events were significantly altered in *ATP13A2* (FS) mutant samples of mDA neurons and progenitor cell line versus the edited wildtype control (with adjusted p-value < 0.1, ΔPSI ≥5). Data for one clonal line is shown as an example. Shown on the y-axis is the number of differential splicing events and difference in percentage spliced in (or ΔPSI) shown on the x-axis. The splicing events were filtered based on the magnitude of delta PSI (ΔPSI). B. Comparison of transcripts from genes that are differentially spliced in 2 different clonal lines of the *ATP13A2* (FS) mutant, which results in 1,181 high confidence differentially spliced gene transcripts. D4 indicates neuronal differentiation set number. C. A graph of Gene Ontology (GO) term enrichment of 1,181 differentially spliced transcripts in *ATP13A2* (FS) mutant protein-expressing mDA neuronal cells compared to the edited wild type control cells. D. Comparison of differentially spliced transcripts in *ATP13A2* (FS) mutant cells to differentially spliced transcripts found in diseased patient brains (Parkinson disease (PD), Parkinson disease with dementia (PDD), dementia with Lewy bodies (DLB)). E. A volcano plot of D4 (differentiation set 4) is shown as an example with some up- and down-regulated gene names shown. DESeq2 analysis of the *ATP13A2* (FS) mutant compared to the edited wild type (EWT) controls reveals 4,209 differentially expressed genes (adjusted p-values < 0.05, FC > 1.5). F. A graph of Gene Ontology (GO) term enrichment of either 2,266 up-regulated genes or 1,943 down-regulated genes in the *ATP13A2* (FS) mutant protein-expressing mDA neuronal cells compared to the edited wild type (EWT) controls.
**Supplemental Figure S9. Differentially spliced (DS) pre-mRNAs between Parkinson disease (PD), Parkinson Disease with dementia (PDD) and Dementia with Lewy bodies (DLB) and control patient brain biopsy (anterior cingulate cortex) data analyzed by either JUM or Leafcutter.** Comparison of PD patient brain cortex data (Feleke et al., 2021) analyzed by either JUM (Wang and Rio, 2018) or Leafcutter (Li et al., 2018). The highest prevalence of differential alternative splicing was observed in patients with Dementia with Lewy Bodies (DLB), by both the JUM and Leafcutter software.
**Supplemental Figure S10. Validations of differential splicing events by reverse transcription-polymerase chain reaction (RT-PCR).** (A) 0.5 μg of total RNA was reverse-transcribed into cDNA according to the manufacturer’s instructions (Bio-Rad, 1708891) and subjected to RT-PCR using primers listed in (B). *UBR4*, *DOCK3*, and *CAMPTA2* are shown as an example from *GBA1* (FS) mut and EWT control cells. *GAPDH* serves as a loading control. (B) Primer information used in RT-PCR validation.

2

## Figures and Tables

**Table:** KEY RESOURCES TABLE

REAGENT or RESOURCE	SOURCE	IDENTIFIER
**Chemicals and Reagents**		
RNeasy Minikit	Qiagen	74104
QIAshredder	Qiagen	79654
NEBNext^®^ Poly(A) mRNA Magnetic Isolation Module	New England Biolabs	E7490
NEBNext Ultra Directional RNA Library Preparation Kit for Illumina	New England Biolabs	E7420S
NEBNext Ultra II Directional RNA Library Prep Kit for Illumina	New England Biolabs	E7760S
NEBNext Multiplex Oligos	New England Biolabs	E7335S, E7500
mTSER-plus Medium	Stem cell Technologies	100–0276
Neurobasal Medium	ThermoFisher Scientific	21103049
B27 Supplement	ThermoFisher Scientific	12587010
N2 Supplement	ThermoFisher Scientific	17502048
L-Glutamine	Sigma	G8450
Penicillin-Streptomycin	ThermoFisher Scientific	15140122
Y-27632 Dihydrochloride Rock inhibitor	Chemdea	CD0141
iMatrix-511	Takara	T304
DPBS (No calcium, No magnesium	ThermoFisher Scientific	SH30028
Accutase	Innovative Cell Technologies	AT104
Matrigel	Corning	354230
Poly L Ornithine	Sigma	P3655
Cultrex Laminin	R&D Systems	3400–010-02
LDN	Stemgent	04–0074
SHH	R&D Systems	1845-SH-100
SB431542	SelleckChem	S1067
CHIR99021	Tocris	4423
GDNF	Peprotech	450–10
BDNF	Peprotech	450–02
Dibutyryl-cAMP (Bucladesine)	Selleckchem	S7858
Sodium L-Ascorbate	Sigma	A40–34
TGFb3	R&D Systems	8420-B3–005
DAPT	Tocris	2634
**Software and Algorithms**		
JUM.2.0.2		Wang and Rio 2018
STAR_2.5.3a		Dobin et al. 2013
HTSeq		Anders et al. 2015
DESeq2		Love et al. 2014
