# Supplementary material for: Alternative pre-mRNA Splicing and Gene Expression Patterns in Midbrain Lineage Cells Carrying Familial Parkinson’s Disease Mutations
Source: bioRxiv. 2025 Sep 4:2024.02.28.582420. Preprint. [Version 4] doi: 10.1101/2024.02.28.582420 (PMC12424998; doi:10.1101/2024.02.28.582420)
Supplement: Supplement 1 — Supplemental Figure S1. Differentially spliced and differentially expressed transcripts in DA neuronal cells expressing the PINK1 (Q129X) mutation. A. Differential alternative splicing events detected upon PINK1 (Q129X) in differentiated DA neurons and progenitor cells (35d) using JUM (Wang and Rio, 2018). 2,905 splicing events were significantly altered in PINK1 (Q129X) mutant samples in DA neurons and progenitor cell line versus the edited wildtype control (with adjusted p-value < 0.1, ΔPSI ≥5). Data for one of three clonal mutant cell lines is shown as an example. Shown on the y-axis is the number of differential splicing events and difference in percentage-spliced-in (or ΔPSI) shown on the x-axis. The splicing events were filtered based on the magnitude of delta PSI (ΔPSI). B. Comparison of genes that are differentially spliced in 3 different clonal lines of PINK1 (Q129X) mutant, this results in 2,905 high confidence differentially spliced genes. D2 indicates different neuronal differentiation set number. C. A graph of Gene Ontology (GO) term enrichment of 2,905 differentially spliced transcripts in PINK1 (Q129X) mutant protein-expressing DA neuronal cells compared to the edited wild type control (EWT). D. Comparison of differentially spliced genes in PINK1 (Q129X) mutant cells to differentially spliced transcripts from genes found in Lewy body disease patient brain biopsy RNA-seq data (Feleke et al., 2021): (Parkinson disease (PD), Parkinson disease with dementia (PDD), dementia with Lewy bodies (DLB)). E. A volcano plot of D2 (differentiation set 2) is shown as an example with some up- and down-regulated gene names shown. DESeq2 analysis of the PINK1 (Q129X) mutant compared to the edited wild type (EWT) controls reveals 7,081 differentially expressed genes (adjusted p-values < 0.05, FC > 1.5). F. A graph of Gene Ontology (GO) term enrichment of either 3,998 up-regulated genes or 3,083 down-regulated genes in the PINK1 (Q129X) mutant protein-expressing mDA neur [file media-1.pdf]

Supplemental Figure S1

A

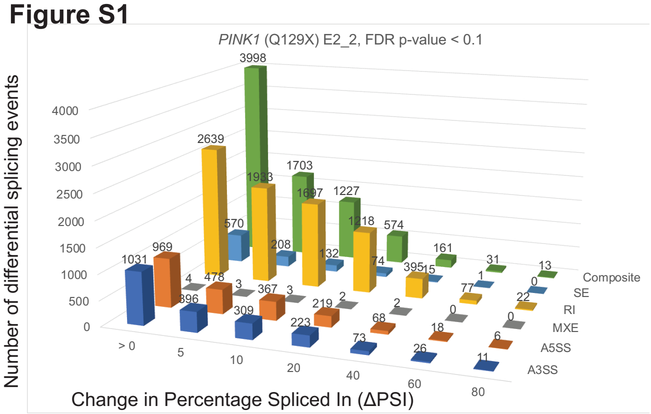

FDR p-value < 0.1, ΔPSI ≥ 5		
PINK1 (Q129X) E2_2		
PSI ≥ 5	Events	geneID
A3SS	396	278
A5SS	478	458
MXE	3	1
RI	1933	809
SE	208	186
Composite	1703	1455
Total	4721	2837

B

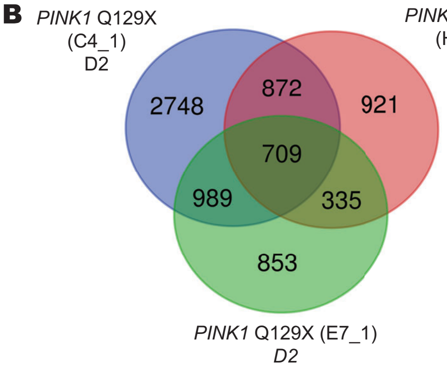

C

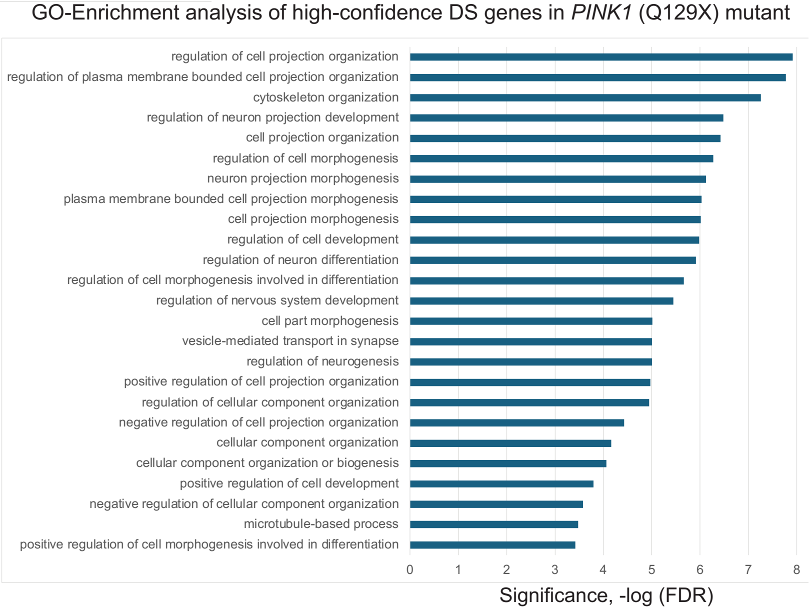

D

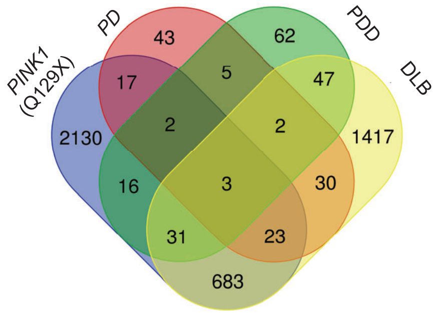

	total	Genes
DLB, PD, PDD, *PINK1* (Q129X)	3	*SEC14L1, SRRM2, EPB41L2*
PD, PDD, *PINK1* (Q129X)	2	*PLXNB2, MEF2BNB-MEF2B*
DLB, PD, *PINK1* (Q129X)	23	*UPF3A, MAST4, WDR27, GRK4, ZNF789, CALD1, UBR4, NRXN2, MACF1, PRUNE2, ABLIM2, MRPL43, EML2, CPNE5, AP3B1, UBE2Q2, KIDINS220, PTPRF, EVL, SLC25A16, CADPS, DOCK10, MICAL2*
DLB, PDD, *PINK1* (Q129X)	31	*NADSYN1, ZFHX4-AS1, TARSL2, CLIP2, LINC-PINT, ERBIN, SGIP1, RBMS2, STRADA, ANK2, UNK, ZDHHC11, ING4, MSI2, DLG1, ZNF718, WIPI2, VEZT, PPP1R32, FAM228B, TARBP1, CTTNBP2, TNRC6A, NPIPA8, COL16A1, ACACB, PDE8A, HSF4, ODF2, NT5C2, FLJ36777*
PD, *PINK1* (Q129X)	17	*FHL1, LRRC16B, PPP6R2, SLIT2, SYNC, SLC12A5, RASGRF2, TENM4, RBFOX1, RYR1, SYT7, TYK2, LINC00403, ZCWPW1, MYO5A, RRP8, CTNNA2*
PDD, *PINK1* (Q129X)	16	*ZNF83, TMEM161B-AS1, MAPK7, LAMA3, CHD2, PLEKHH1, UBAP2, INPP1, ANK1, UGP2, LARS2, EOGT, DPY19L1P1, TMEM91, ELP2, C3orf14*

E

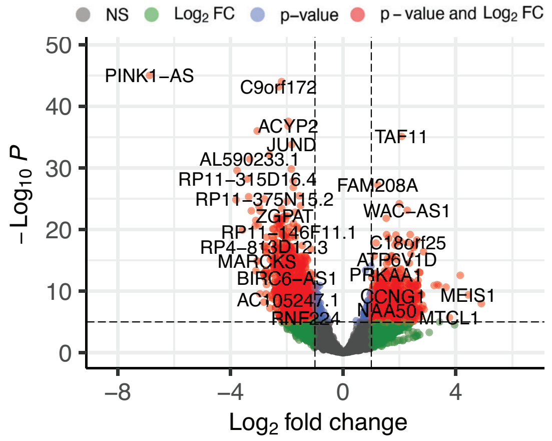

F

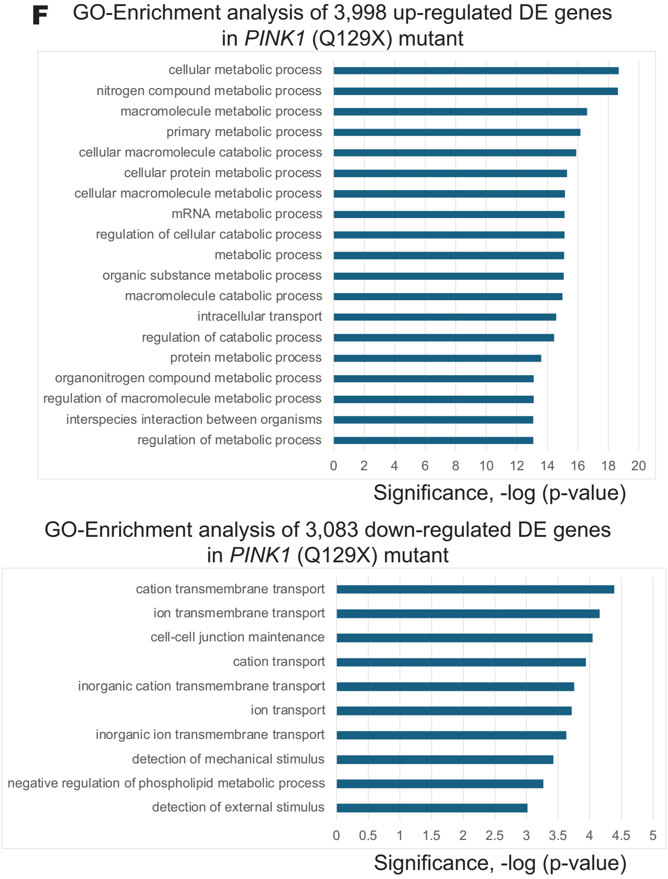

Supplemental Figure S2

A

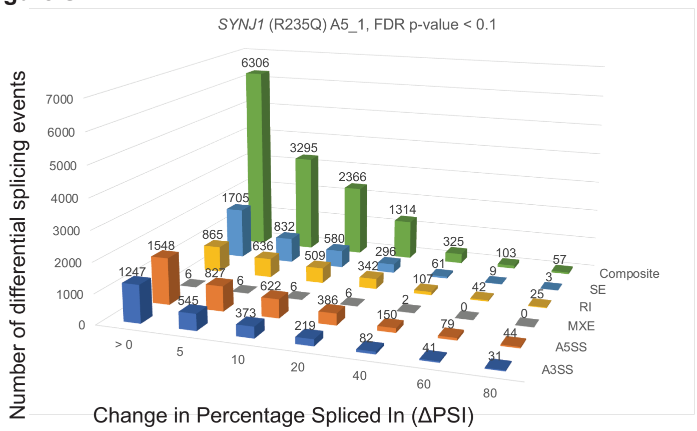

FDR p-value < 0.1, △PSI ≥ 5		
SYNJ1 (R235Q) A5_1		
PSI >5	Events	geneID
A3SS	545	441
A5SS	827	714
MXE	6	5
RI	636	522
SE	832	751
Composite	3295	2751
Total	6141	4481

B

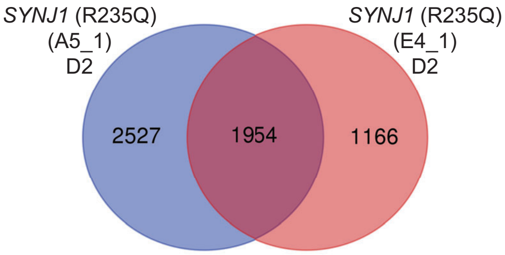

GO-Enrichment analysis of 1,954 high-confidence DS genes in *SYNJ1* (R235Q) mutant

C

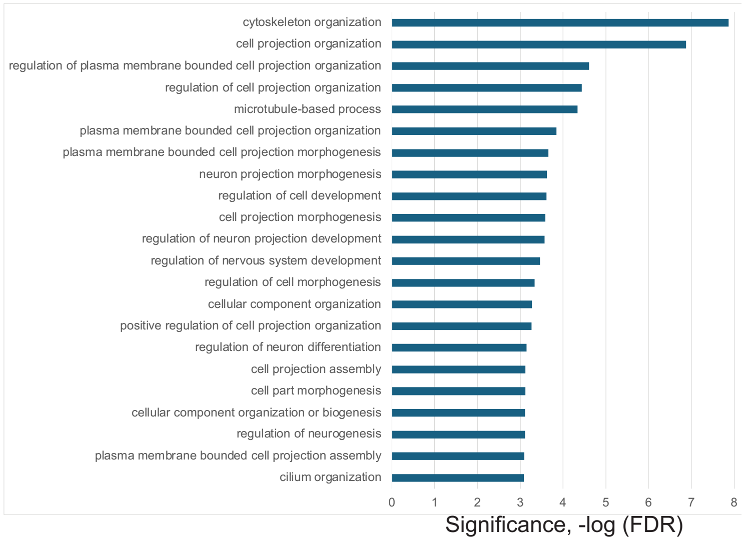

D

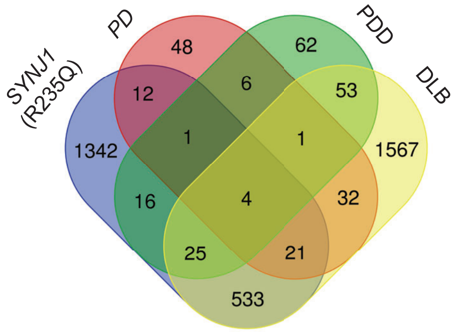

	total	Genes
DLB, PD, PDD, *SYNJ1* (R235Q)	4	*SEC14L1, SRRM2, EPB41L2, SEC24C*
PD, PDD, *SYNJ1* (R235Q)	1	*MEF2BNB-MEF2B*
DLB, PD, *SYNJ1* (R235Q)	21	*CPNE5, AP3B1, FYN, MAST4, KIDINS220, PTPRF, CAPS2, GRK4, KIF3A, ZNF789, CALD1, EVL, MACF1, SLC25A16, CADPS, CD46, AK2, MRPL43, EML2, DENND3, MICAL2*
DLB, PDD, *SYNJ1* (R235Q)	25	*DLG1, FBXO42, ZNF718, ZFHX4-AS1, WIPI2, VEZT, CLIP2, PPP1R32, LINC-PINT, FAM228B, TARBP1, MYO9A, COL16A1, SHTN1, ARHGAP17, STX2, STRADA, AP4B1, ANK2, HSF4, TRIM9, DYNC2H1, ODF2, FAM49B, ST18*
PD, *SYNJ1* (R235Q)	12	*RBFOX1, RYR1, FHL1, FMNL1, TYK2, ZNF91, SLIT2, PSD3, ZCWPW1, RRP8, ZNF708, RASGRF2*
PDD, *SYNJ1* (R235Q)	16	*UBE2F-SCLY, ZNF83, TMEM161B-AS1, MITD1, CCDC113, MAPK7, PLEKHH1, POLR2J3, ELP2, MAP7D2, C4orf33, DOT1L, ARHGAP18, ANK1, KIAA1191, TCEA3*

E

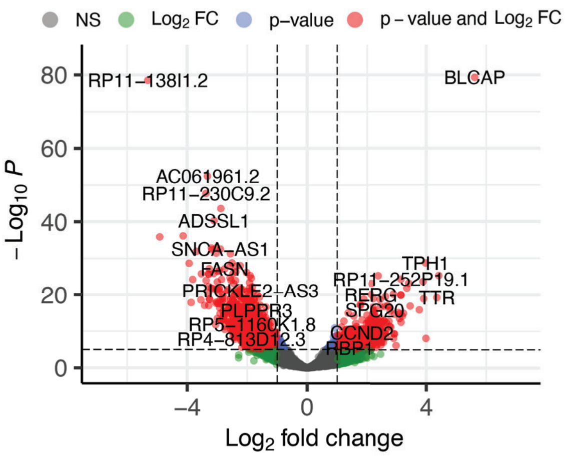

F

GO-Enrichment analysis of 3,211 up-regulated DE genes in *SYNJ1* (R258Q) mutant

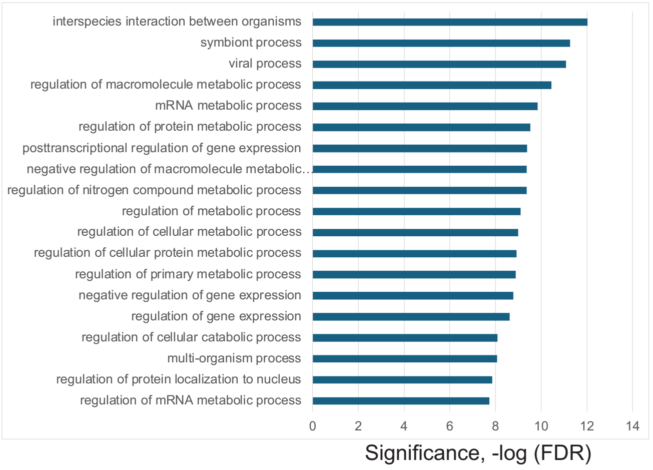

GO-Enrichment analysis of 3,093 down-regulated DE genes in *SYNJ1* (R258Q) mutant

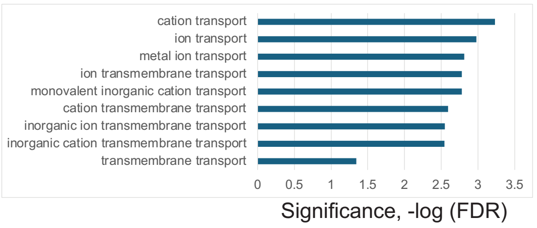

Supplemental Figure S3

A

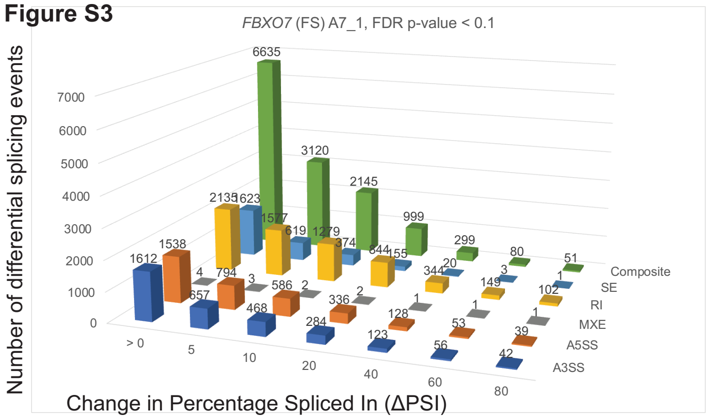

FDR p-value < 0.1, ΔPSI ≥ 5		
FBXO7 (FS) A7_1		
PSI >5	Events	geneID
A3SS	657	532
A5SS	794	701
MXE	3	2
RI	1577	1198
SE	619	558
Composite	3120	2648
Total	6770	4752

B

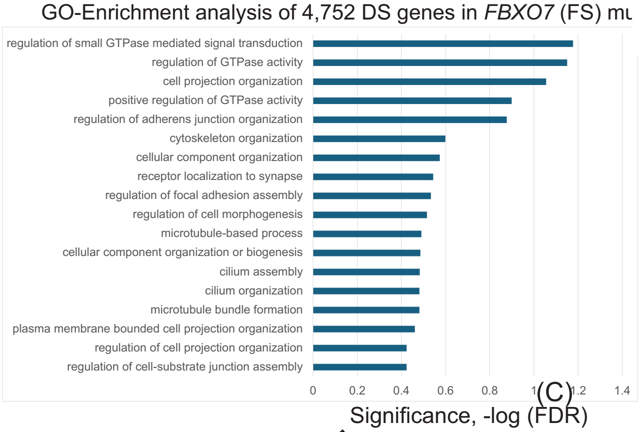

D

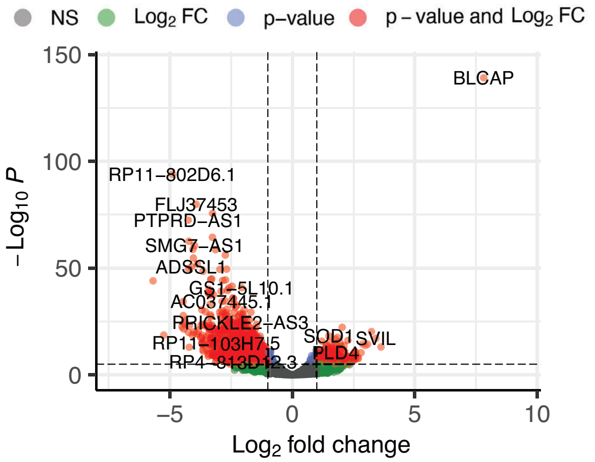

C

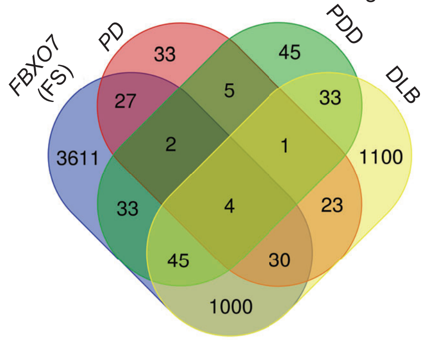

	total	Genes
DLB, *FBXO7* (FS), PD, PDD	4	*SEC14L1, ADHFE1, SRRM2, EPB41L2*
FBXO7 (FS), PD, PDD	2	*MEF2BNB-MEF2B, ZGPAT*
DLB, *FBXO7* (FS), PD	30	*SNX19, UPF3A, MAST4, EML4, WDR27, GRK4, ZNF789, CALD1, UBR4, C1orf162, NRXN2, MACF1, PRUNE2, ABLIM2, EML2, B3GALNT1, CPNE5, AP3B1, FYN, UBE2Q2, KIDINS220, PTPRF, EVL, L3MBTL1, LPGAT1, SLC25A16, CD46, DENND3, MICAL2, LRP2BP*
DLB, *FBXO7* (FS), PDD	45	*NADSYN1, ZFHX4-AS1, ACAP3, TARSL2, LINC-PINT, BBS9, MYO9A, USP33, ERBIN, SHTN1, SLTM, ARHGAP17, RBMS2, STRADA, AP4B1, ANK2, UNK, DYNC2H1, ZDHHC11, ING4, MSI2, ROM1, DLG1, FBXO42, ZNF718, VEZT, PPP1R32, FAM228B, TARBP1, CTTNBP2, TNRC6A, NPIPA8, SYTL2, COL16A1, LUC7L3, MPRIP, STX2, PDE8A, HSF4, TRIM9, NT5C2, PEX5L, NPL, FAM49B, ST18*
FBXO7 (FS), PD	27	*PPP6R2, IKBKAP, SLIT2, PSD3, ANKZF1, SLC12A5, ARHGAP6, RELN, RASGRF2, TENM4, BBS7, WDR59, WBSCR22, RYR1, SYT7, TXNL4A, GABRA1, PLA2G7, ZNF91, LINC00403, ETV1, ZCWPW1, MYO5A, RRP8, SCN2A, ZNF708, CYB561D1*
FBXO7 (FS), PDD	33	*HAUS2, PDLIM2, ZNF83, TMEM161B-AS1, MAPK7, CHD2, PLEKHH1, MKL2, TMEM218, POLR2J3, PANK2, POLG2, MAP7D2, TIGD7, PREB, XPO1, ANK1, KIAA1191, UGP2, STARD9, LOC100130950, DCAF8, UBE2F-SCLY, SPG11, TNFSF12, MITD1, DPY19L1P1, TMEM91, LINC01170, TIRAP, RNF8, DROSHA, DOT1L*

F

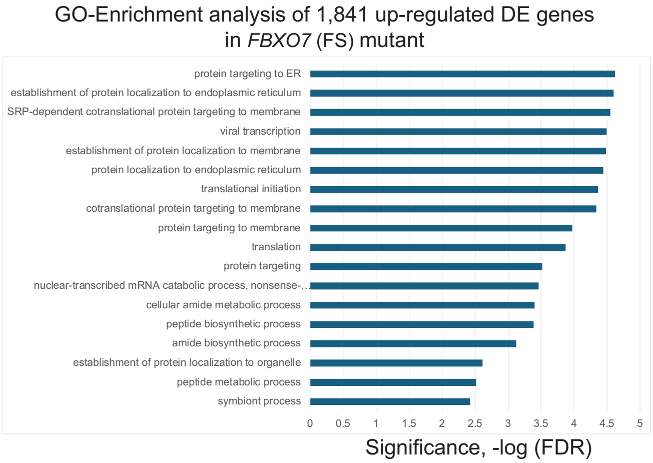

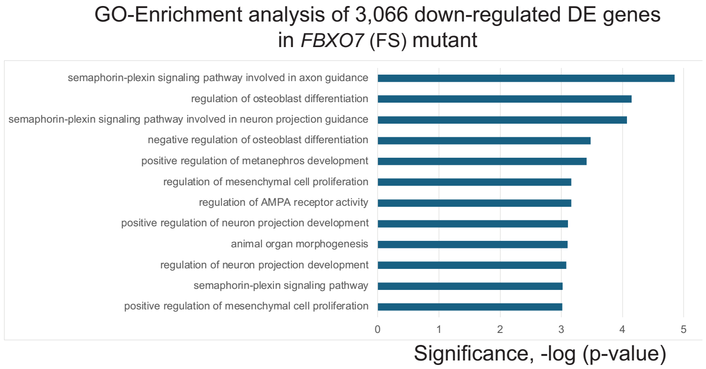

Supplemental Figure S4

A

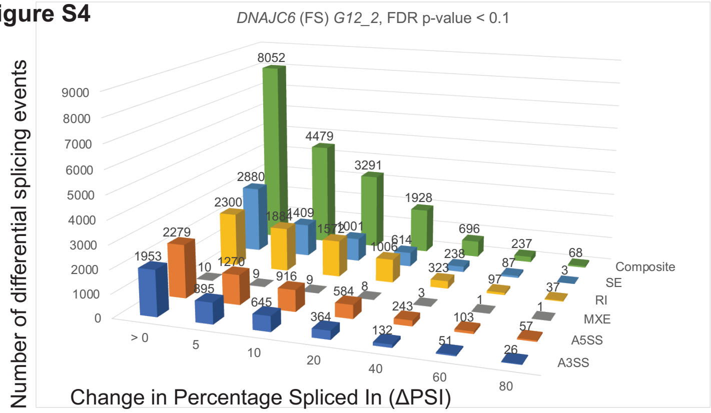

FDR p-value < 0.1, ΔPSI ≥ 5		
DNAJC6 (FS) G12_2		
PSI >5	Events	geneID
A3SS	895	782
A5SS	1270	1087
MXE	9	9
RI	1884	1256
SE	1409	1202
Composite	4479	3610
Total	9946	6084

B

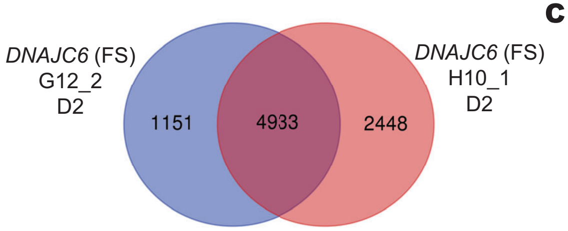

C GO-Enrichment analysis of 4,933 high-confidence DS genes in *DNAJC6* (FS) mutant

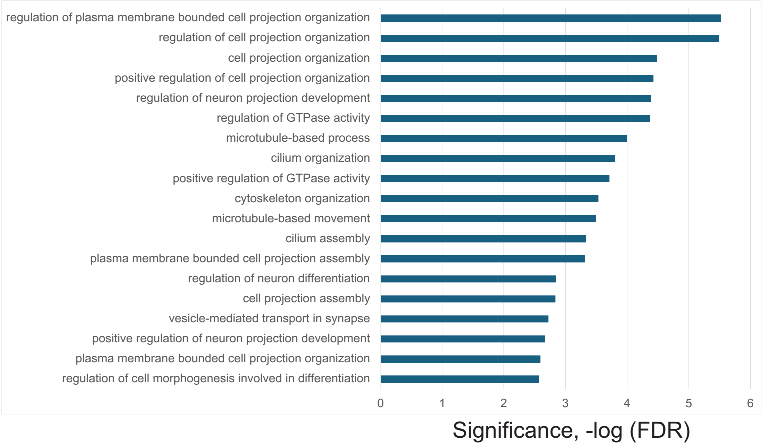

D

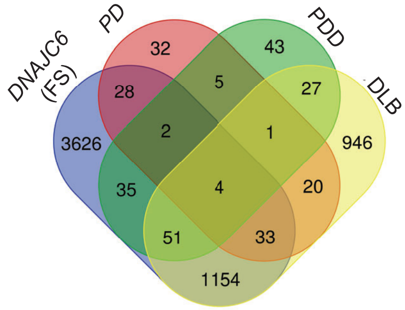

	total	Genes
DLB, *DNAJC6* (FS), PD, PDD	4	*SEC14L1, SEC24C, SRRM2, EPB41L2*
DNAJC6 (FS), PD, PDD	2	*PLXNB2, MEF2BNB-MEF2B*
DLB, *DNAJC6* (FS), PD	33	*SNX19, UPF3A, MAST4, EML4, WDR27, GRK4, KIF3A, ZNF789, CALD1, UBR4, MACF1, PRUNE2, ABLIM2, MRPL43, CPNE5, TMEM99, AP3B1, FYN, UBE2Q2, KIDINS220, PTPRF, GABRG2, EVL, L3MBTL1, LPGAT1, CADPS, CD46, AK2, APIP, DENND3, DOCK10, MICAL2, LRP2BP*
DLB, *DNAJC6* (FS), PDD	51	*NADSYN1, ZFHX4-AS1, TARSL2, CLIP2, LINC-PINT, STK39, BBS9, MYO9A, USP33, ERBIN, SHTN1, SLTM, ARHGAP17, SGIP1, RBMS2, STRADA, ANK2, UNK, DYNC2H1, TPRA1, ZDHHC11, DCUN1D4, MSI2, ROM1, DLG1, FBXO42, ZNF718, BRD8, WIPI2, VEZT, PPP1R32, FAM228B, TARBP1, TNRC6A, IKBKB, SYTL2, LUC7L3, MPRIP, MICU3, STX2, PDE8A, HSF4, TRIM9, INO80C, ODF2, ATP5SL, NT5C2, PEX5L, FAM49B, ST18, PIK3IP1*
DNAJC6 (FS), PD	28	*FHL1, TCFL5, FMNL1, ZCCHC2, DUS1L, SLIT2, PSD3, ANKZF1, SYNC, VARS2, SLC12A5, ARHGAP6, RASGRF2, TENM4, RBFOX1, WBSCR22, RYR1, SYT7, TYK2, ZNF91, LINC00403, ETV1, ZCWPW1, MYO5A, RRP8, SCN2A, CYB561D1, CTNNA2*
DNAJC6 (FS), PDD	35	*ZNF83, GFPT2, TMEM161B-AS1, MAPK7, CHD2, PLEKHH1, MKL2, TMEM218, UBAP2, POLG2, ZFHX4, MAP7D2, C4orf33, TIGD7, ARHGAP18, PREB, OGFOD3, XPO1, ANK1, KIAA1191, UGP2, STARD9, EOGT, NPC1, DCAF8, UBE2F-SCLY, ALDH3A2, DRAM2, SPG11, CCDC113, DPY19L1P1, TMEM91, ELP2, ESD, DOT1L*

E

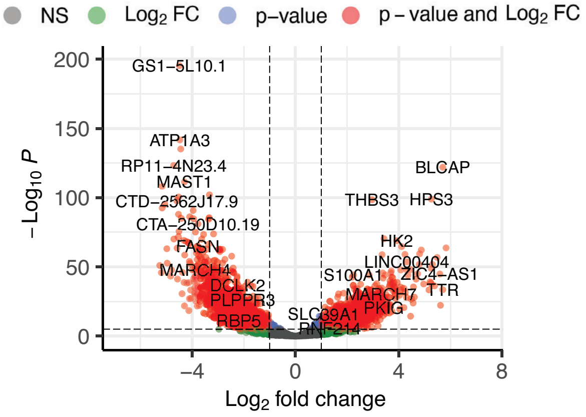

F

GO-Enrichment analysis of 3,763 up-regulated DE genes in *DNAJC6* (FS) mutant

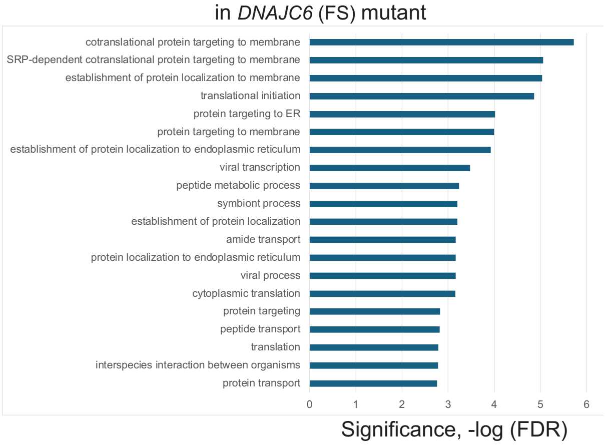

GO-Enrichment analysis of 3,620 down-regulated DE genes in *DNAJC6* (FS) mutant

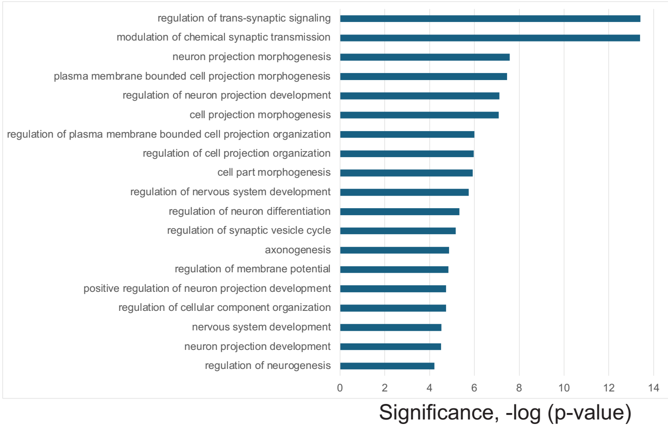

Supplemental Figure S5

A

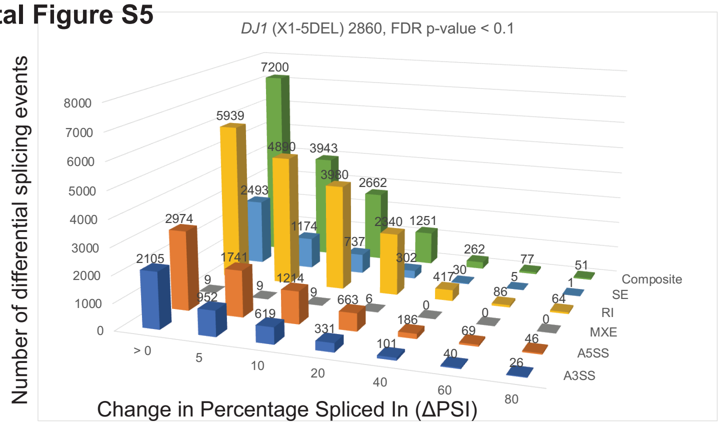

FDR p-value < 0.1, ΔPSI ≥ 5		
DJ1 (X1-5DEL) 2860		
PSI >5	Events	geneID
A3SS	952	784
A5SS	1741	1530
MXE	9	8
RI	4890	3047
SE	1174	1044
Composite	3943	3229
Total	12709	7201

B

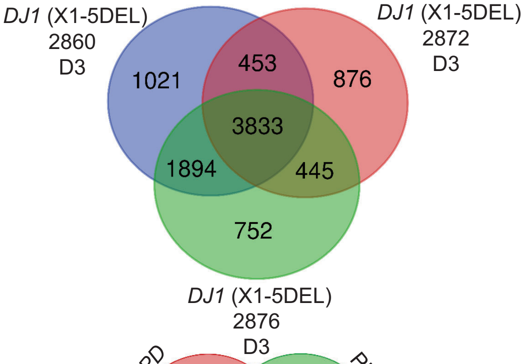

C

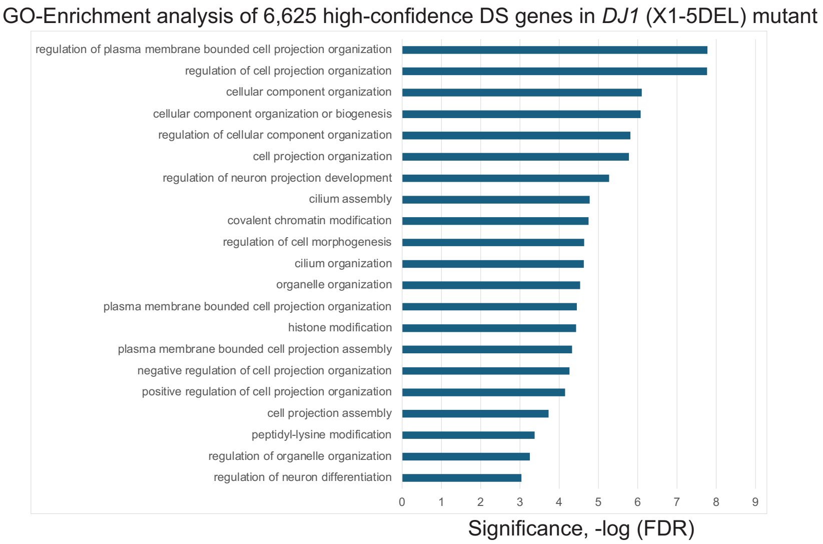

D

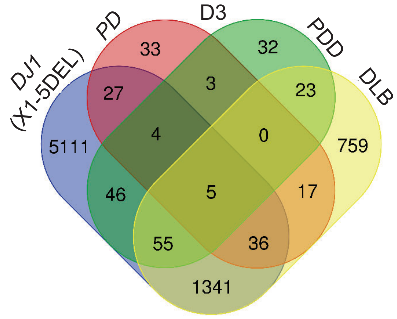

	total	Genes
DJ1 (X1-5DEL), DLB, PD, PDD	5	*SEC14L1, ADHFE1, SEC24C, SRRM2, EPB41L2*
DJ1 (X1-5DEL), PD, PDD	4	*PLXNB2, MEF2BNB-MEF2B, DDX46, CCDC93*
DJ1 (X1-5DEL), DLB, PD	36	*SNX19, UPF3A, ABHD12, MAST4, EML4, WDR27, GRK4, KIF3A, ZNF789, CALD1, UBR4, NRXN2, MACF1, PRUNE2, ABLIM2, MRPL43, EML2, B3GALNT1, TCTN2, FYN, SPIDR, UBE2Q2, KIDINS220, PTPRF, CAPS2, EVL, L3MBTL1, SCN3B, SLC25A16, CADPS, CD46, AK2, DOCK10, MICAL2, VCL, LRP2BP*
DJ1 (X1-5DEL), DLB, PDD	55	*NADSYN1, ZFHX4-AS1, ACAP3, TARSL2, CLIP2, LINC-PINT, STK39, SLC16A4, USP33, SHTN1, SLTM, ARHGAP17, SGIP1, FAM171A1, STRADA, AP4B1, ANK2, UNK, TPRA1, ZDHHC11, DCUN1D4, ING4, MSI2, ROM1, DLG1, FBXO42, BRD8, VEZT, PPP1R32, FAM228B, TARBP1, TNRC6A, IKBKB, SYTL2, COL16A1, TMEM14B, LUC7L3, ACACB, MPRIP, MICU3, STX2, PDE8A, HSF4, TRIM9, INO80C, ODF2, ATP5SL, OPTN, PRPF18, NT5C2, FLJ36777, PEX5L, NPL, FAM49B, ST18*
DJ1 (X1-5DEL), PD	27	*FHL1, LRRC16B, TCFL5, PPP6R2, IKBKAP, FMNL1, C19orf18, DUS1L, SLIT2, PSD3, ANKZF1, SYNC, SLC12A5, RASGRF2, WDR59, RBFOX1, WBSCR22, ZNF781, SYT7, TYK2, DCHS2, ZCWPW1, TAMM41, MYO5A, NSUN2, RRP8, CYB561D1*
DJ1 (X1-5DEL), PDD	46	*HAUS2, ZNF83, GFPT2, TMEM161B-AS1, NOXA1, MAPK7, LAMA3, CHD2, PLEKHH1, MKL2, TMEM218, POLR2J3, PANK2, UBAP2, POLG2, CYP4V2, UBR5, INPP1, TIGD7, ARHGAP18, PREB, OGFOD3, XPO1, ANK1, GLYR1, KIAA1191, EOGT, NPC1, DCAF8, UBE2F-SCLY, ALDH3A2, DRAM2, SPG11, KIAA0586, SDHAF2, TMEM91, TIRAP, ELP2, C3orf14, CACNG7, ESD, DROSHA, DOT1L, TCEA3, GPR89A, SNX17*

E

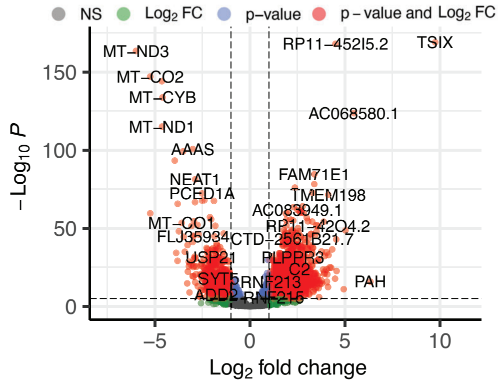

F

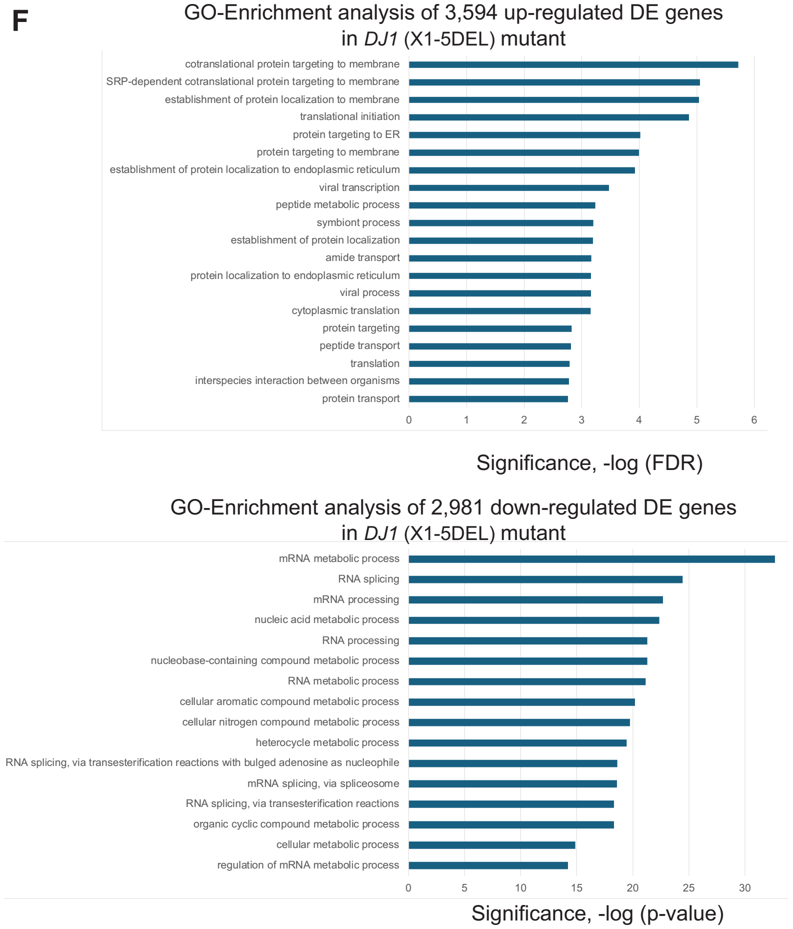

Supplemental Figure S6

A

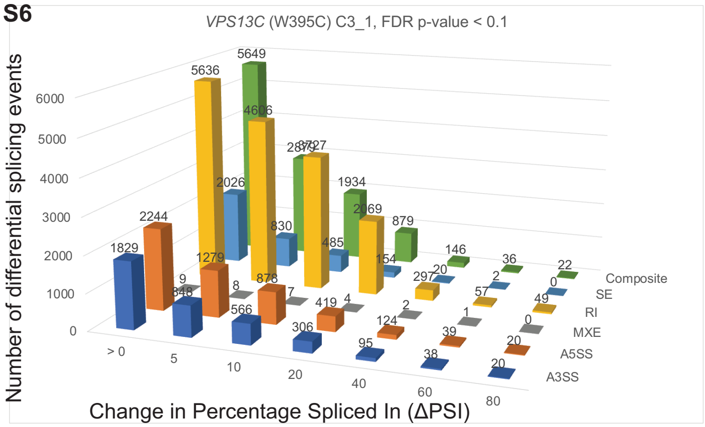

FDR p-value < 0.1, ΔPSI ≥ 5		
VPS13C (W395C) C3_1		
PSI >5	Events	geneID
A3SS	848	715
A5SS	1279	1135
MXE	8	8
RI	4606	2979
SE	830	759
Composite	2879	2440
Total	10450	6230

B

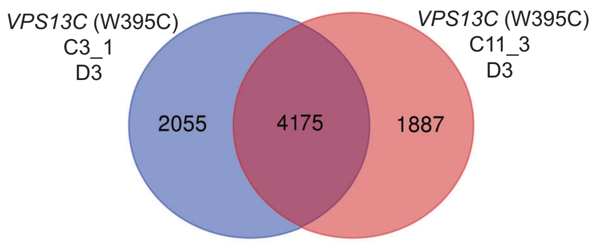

C GO-Enrichment analysis of 4,175 high-confidence DS genes in *VPS13C* (W395C) mutant

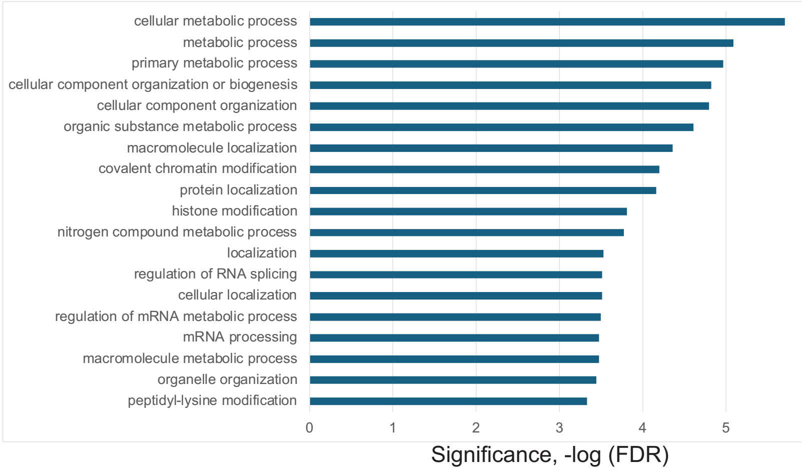

D

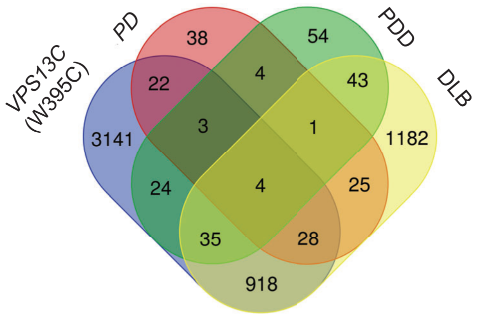

	total	Genes
DLB, PD, PDD, *VPS13C* (W395C)	4	*SEC14L1, SEC24C, SRRM2, EPB41L2*
PD, PDD, *VPS13C* (W395C)	3	*PLXNB2, MEF2BNB-MEF2B, DDX46*
DLB, PD, *VPS13C* (W395C)	28	*SNX19, UPF3A, ABHD12, MAST4, EML4, WDR27, GRK4, ZNF789, CALD1, UBR4, NRXN2, MACF1, PRUNE2, ABLIM2, EML2, B3GALNT1, TCTN2, FYN, SPIDR, PTPRF, SCN3B, SLC25A16, CADPS, CD46, AK2, MICAL2, VCL, LRP2BP*
DLB, PDD, *VPS13C* (W395C)	35	*NADSYN1, ZFHX4-AS1, ACAP3, SLC16A4, USP33, SLTM, ARHGAP17, SGIP1, FAM171A1, AP4B1, ANK2, UNK, TPRA1, ZDHHC11, DCUN1D4, ING4, ROM1, DLG1, FBXO42, BRD8, PPP1R32, FAM228B, TARBP1, TNRC6A, IKBKB, COL16A1, TMEM14B, LUC7L3, ACACB, PDE8A, HSF4, INO80C, ODF2, OPTN, FAM49B*
PD, *VPS13C* (W395C)	22	*FHL1, LRRC16B, TCFL5, PPP6R2, IKBKAP, DUS1L, SLIT2, ANKZF1, SYNC, SLC12A5, ECD, RBFOX1, WBSCR22, TXNL4A, TYK2, ZNF91, LINC00403, DCHS2, TAMM41, MYO5A, NSUN2, RRP8*
PDD, *VPS13C* (W395C)	24	*TMEM161B-AS1, MAPK7, CHD2, MKL2, TMEM218, UBAP2, POLG2, CYP4V2, INPP1, ARHGAP18, PREB, XPO1, GLYR1, STARD9, NPC1, DCAF8, ALDH3A2, DRAM2, KIAA0586, TMEM91, DROSHA, DOT1L, GPR89A, SNX17*

GO-Enrichment analysis of 3,249 up-regulated DE genes in *VPS13C* (W395C) mutant

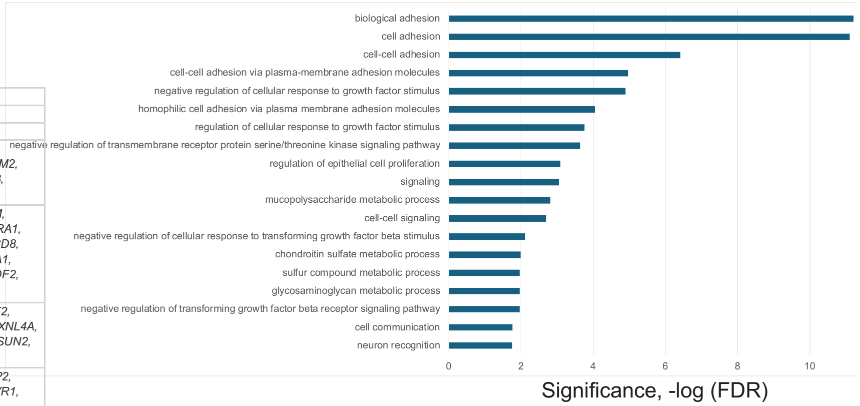

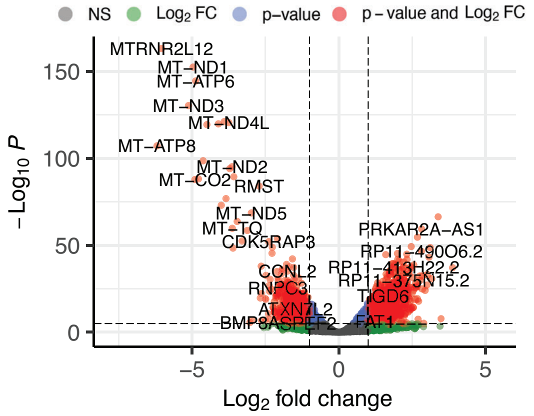

GO-Enrichment analysis of 2,830 down-regulated DE genes in *VPS13C* (W395C) mutant

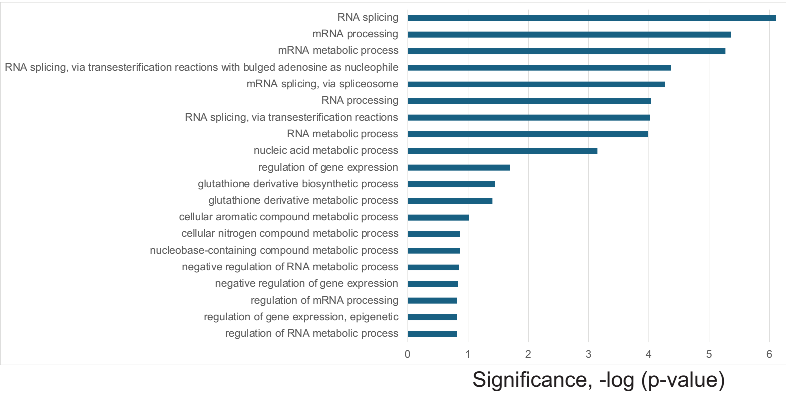

Supplemental Figure S7

A

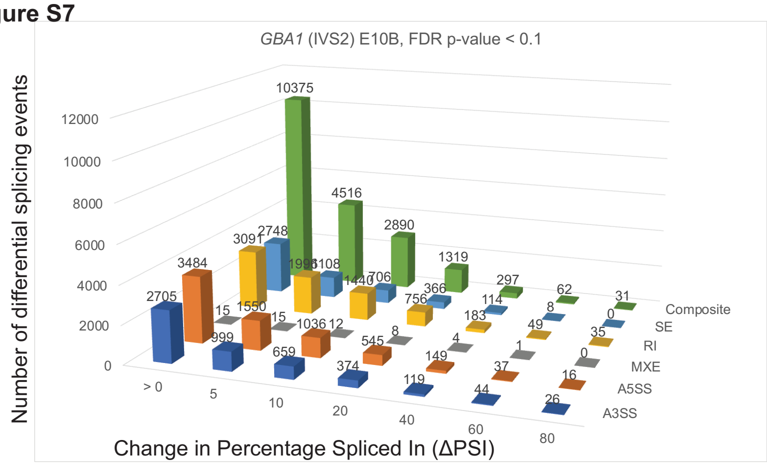

FDR p-value < 0.1, △PSI ≥ 5		
GBA1 (IVS2) E10B		
PSI >5	Events	geneID
A3SS	999	736
A5SS	1550	1269
MXE	15	13
RI	1996	1415
SE	1108	918
Composite	4516	3510
Total	10184	6107

B

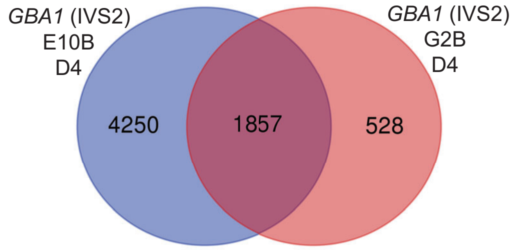

C

GO-Enrichment analysis of 1,857 high-confidence DS genes in *GBA1* (IVS2) mutant

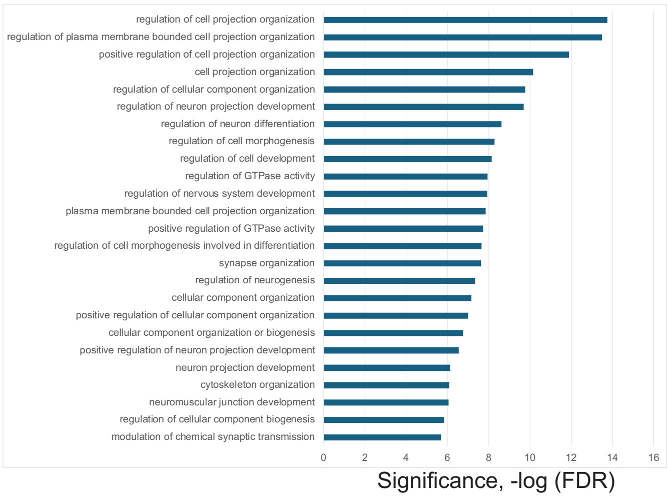

D

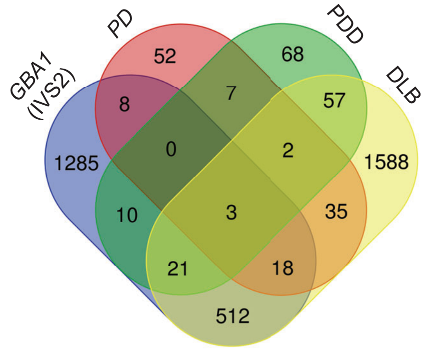

	total	Genes
DLB, *GBA1 (IVS2)*, PD, PDD	3	*SEC14L1, SRRM2, EPB41L2*
DLB, *GBA1* (IVS2), PD	18	*MAST4, KIDINS220, PTPRF, EML4, KIF3A, ZNF789, UBR4, EVL, NRXN2, L3MBTL1, MACF1, CADPS, CD46, PRUNE2, ABLIM2, DOCK10, MICAL2, LRP2BP*
DLB, *GBA1* (IVS2), PDD	21	*DLG1, ZFHX4-AS1, TARSL2, CLIP2, TARBP1, MYO9A, TNRC6A, COL16A1, SHTN1, MPRIP, ARHGAP17, STX2, SGIP1, ANK2, HSF4, INO80C, ZDHHC11, ATP5SL, ROM1, NPL, FAM49B*
GBA1 (IVS2), PD	8	*TENM4, RBFOX1, RYR1, FHL1, TXNL4A, DCHS2, MYO5A, SLC12A5*
GBA1 (IVS2), PDD	10	*UBE2F-SCLY, KIAA0586, LAMA3, TMEM218, POLR2J3, UBAP2, ZFHX4, MAP7D2, XPO1, DCAF8*

E

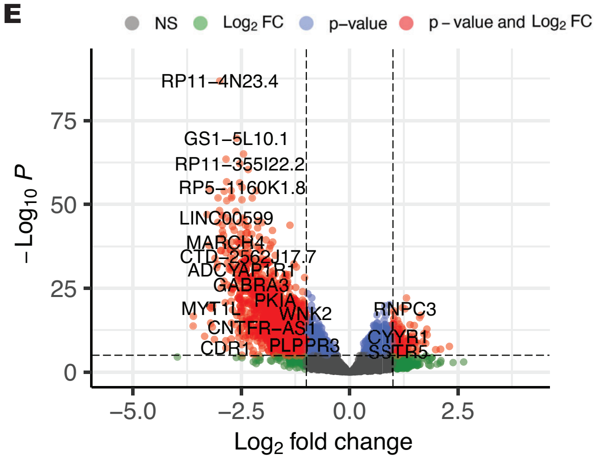

F

GO-Enrichment analysis of 1,615 up-regulated DE genes in *GBA1* (IVS2) mutant

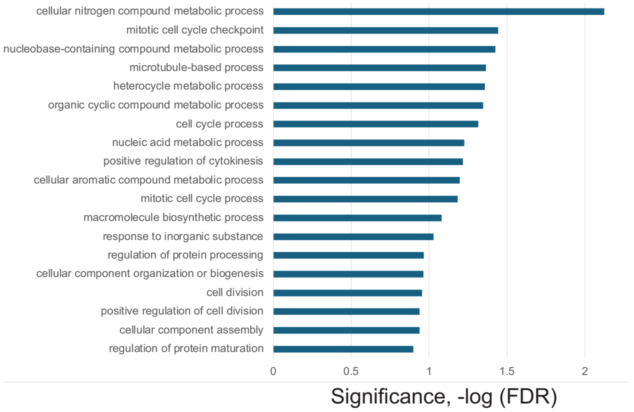

GO-Enrichment analysis of 2,665 down-regulated DE genes in *GBA1* (IVS2) mutant

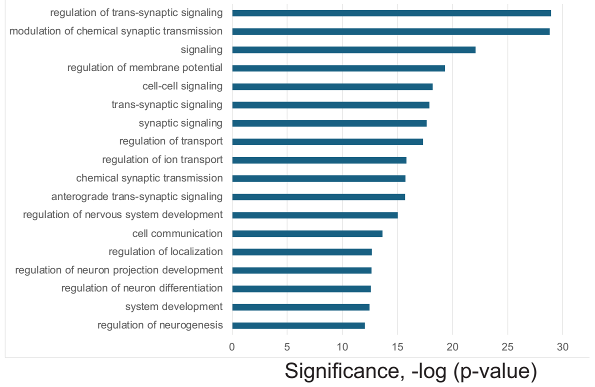

Supplemental Figure S8

A

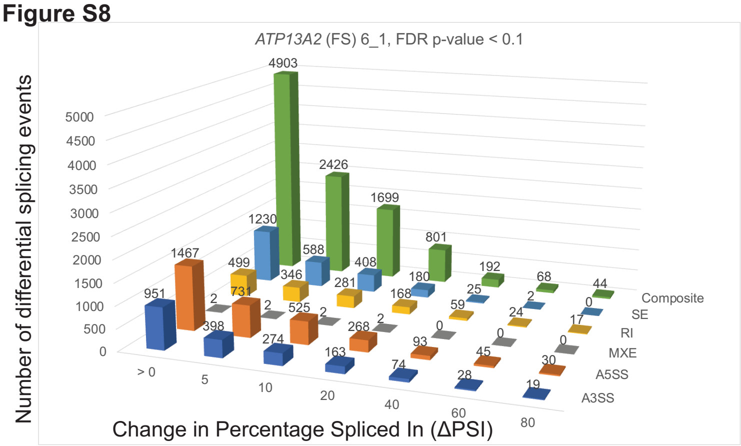

FDR p-value < 0.1, ΔPSI ≥ 5		
ATP13A2 (FS) 6_1		
PSI >5	Events	geneID
A3SS	398	320
A5SS	731	634
MXE	2	2
RI	346	282
SE	588	536
Composite	2426	2056
Total	4491	3380

B

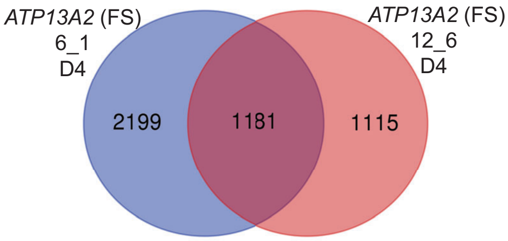

GO-Enrichment analysis of 1,181 high-confidence DS genes in *ATP13A2* (FS) mutant

C

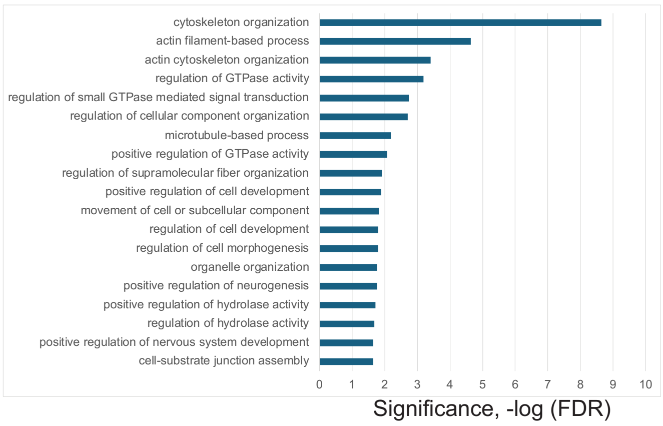

D

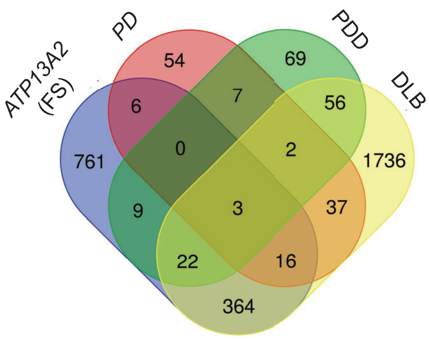

	total	Genes
ATP13A2 (FS), DLB, PD, PDD	3	*SEC14L1, SRRM2, EPB41L2*
ATP13A2 (FS) , DLB, PD	16	*UPF3A, MAST4, KIDINS220, PTPRF, EML4, WDR27, KIF3A, CALD1, L3MBTL1, MACF1, CD46, AK2, ABLIM2, EML2, IPO11, LRP2BP*
ATP13A2 (FS) , DLB, PDD	22	*DLG1, ZFHX4-AS1, VEZT, CLIP2, PPP1R32, TARBP1, CTTNBP2, TNRC6A, ERBIN, COL16A1, SHTN1, MPRIP, ARHGAP17, STX2, STRADA, PDE8A, ANK2, TRIM9, DYNC2H1, INO80C, ATP5SL, NT5C2*
ATP13A2 (FS) , PD	6	*TENM4, RBFOX1, FHL1, SYNC, RRP8, RASGRF2*
ATP13A2 (FS) , PDD	9	*DPY19L1P1, SDHAF2, XRN1, TMEM218, MAP7D2, ESD, C4orf33, SNX17, DCAF8*

E

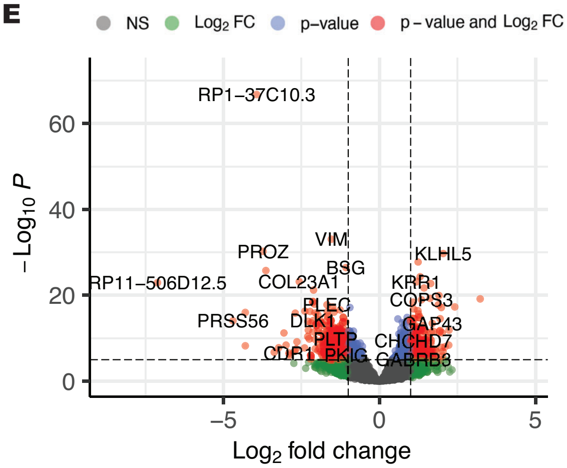

F

GO-Enrichment analysis of 2,266 up-regulated DE genes in *ATP13A2* (FS) mutant

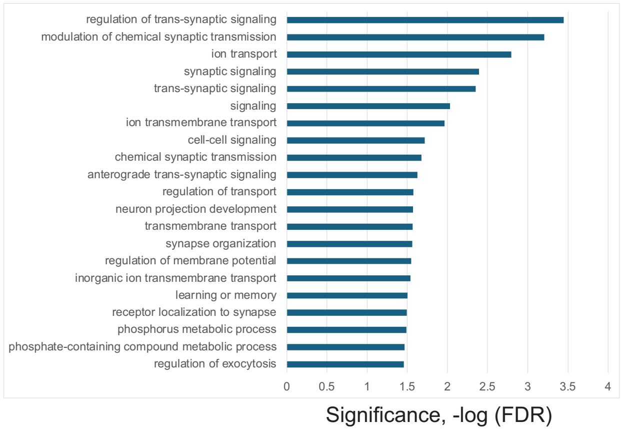

GO-Enrichment analysis of 1,943 down-regulated DE genes in *ATP13A2* (FS) mutant

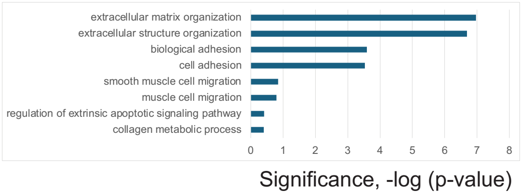

Supplemental Figure S9

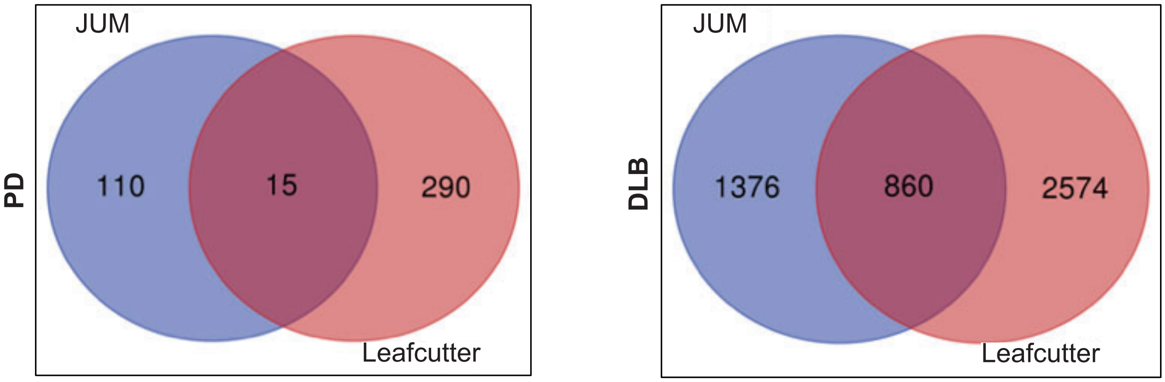

	JUM	Leafcutter
**PD**	125	305
**PDD**	168	357
**DLB**	2,236	3,434

Supplemental Figure S9. Differentially spliced (DS) pre-mRNAs between Parkinson disease (PD), Parkinson Disease with dementia (PDD) and Dementia with Lewy bodies (DLB) and control patient brain biopsy (anterior cingulate cortex) data analyzed by either JUM or Leafcutter. Comparison of PD patient brain cortex data (Feleke et al., 2021) analyzed by either JUM (Wang and Rio, 2018) or Leafcutter (Li et al., 2018). The highest prevalence of differential alternative splicing was observed in patients with Dementia with Lewy Bodies (DLB), by both the JUM and Leafcutter software.

Supplemental Figure S10

(A)

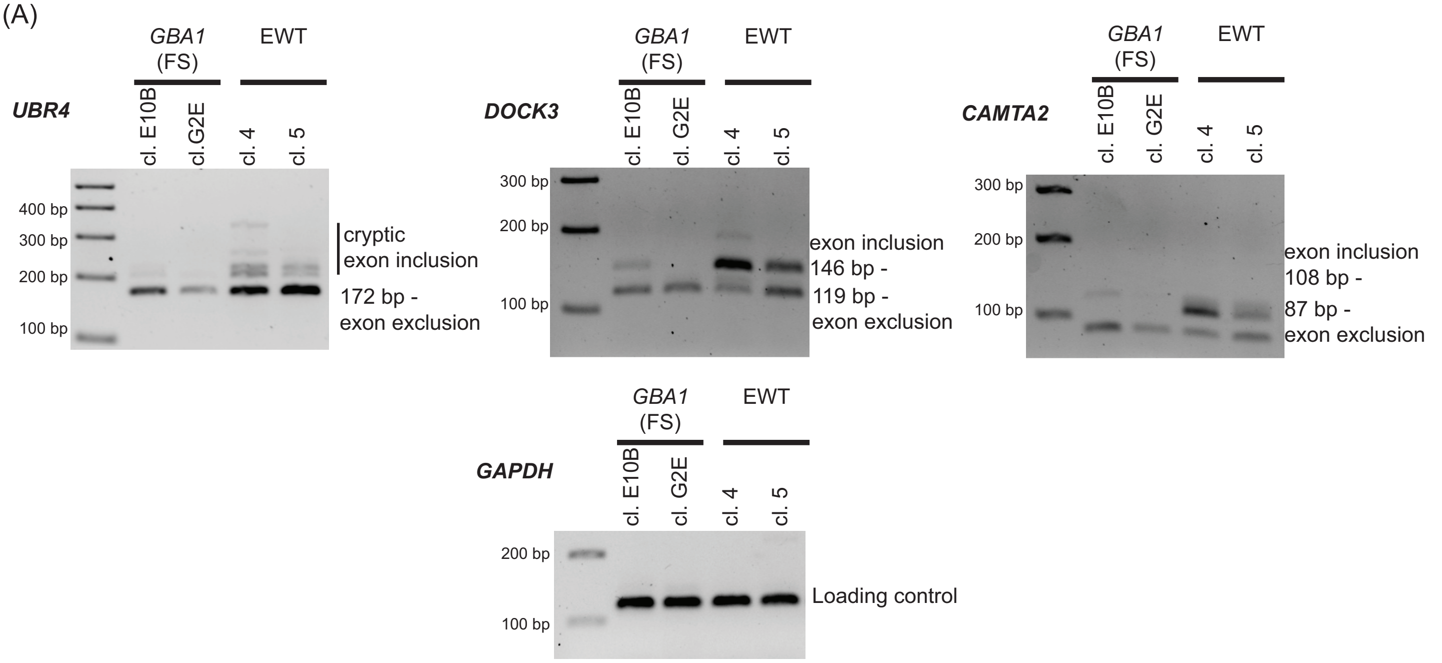

(B)

Primers		Genomic Coordinates (hg38):	ΔPSI (mut-EWT)
UBR4-F	ccttctgcctctgtcagcaa	chr1:19148626-19150575	-0.35 [GBA1 (FS) E10B]<br>-0.29 [GBA1 (FS) G2E]
UBR4-R	tctcgatgattgggccaacg		
DOCK3-F	ggaggccgagttgattgaca	chr3:51330223-51333156	-0.59 [GBA1 (FS) E10B]<br>-0.47 [GBA1 (FS) G2E]
DOCK3-R	cgcgccatgtttcttgttca		
CAMTA2-F	cagcgctgttaccggaagta	chr17:4969337-4969628	-0.58 [GBA1 (FS) E10B]<br>-0.36 [GBA1 (FS) G2E]
CAMTA2-R	gcttcggaacttgctctgga		
GAPDH-F	ctctgctcctcctgttcgac		
GAPDH-R	gcgcccaatacgaccaaatc		

Supplemental Figure S10. Validations of differential splicing events by RT-PCR. (A) 0.5 µg of total RNA was reverse-transcribed according to the manufacturer's instructions (Bio-Rad, 1708891) and subjected to RT–PCR using primers listed in (B). *UBR4*, *DOCK3*, and *CAMPTA2* are shown as an example from *GBA1* (FS) mut and EWT control cells. *GAPDH* serves as a loading control. (B) Primer information used in RT-PCR validation.
