# Supplementary material for: Alternative pre-mRNA Splicing and Gene Expression Patterns in Midbrain Lineage Cells Carrying Familial Parkinson’s Disease Mutations
Source: bioRxiv. 2025 Sep 4:2024.02.28.582420. Preprint. [Version 4] doi: 10.1101/2024.02.28.582420 (PMC12424998; doi:10.1101/2024.02.28.582420)
Supplement: 2 [file NIHPP2024.02.28.582420v4-supplement-2.pdf]

Supplemental Table S1. Differentially spliced gene transcripts in familial PD mutant cells
Supplemental Table S2. Summary of genes and GO enrichment analysis for differential splicing analysis
Supplemental Table S3. Summary of genes and GO enrichment analysis for differential expression profiling
Supplemental Table S4. Genomic Coordinates of Detected Splicing Junctions by JUM - *DOCK10*
Supplemental Table S5. Genomic Coordinates of Detected Splicing Junctions by JUM - *SRRM2*
Supplemental Table S6. Comparison of differentially spliced genes and differentially expressed genes
